# Association of mental health status between self-poisoning suicide patients and their family members: a matched-pair analysis

**DOI:** 10.1186/s12888-023-04779-9

**Published:** 2023-04-28

**Authors:** Wenjing Zheng, Limei Han, Yanna Fan, Min Yi, Xiaoxia Lu, Juan Yang, Xiaobo Peng, Ying Yang

**Affiliations:** 1 Department of Chemical Poisoning Treatment, Senior Department of Hematology, Fifth Medical Center of PLA General Hospital, Beijing, China; 2 Department of Radiotherapy, Senior Department of Oncology, Fifth Medical Center of PLA General Hospital, Beijing, China; 3 Institute of Medical Information and Library, Chinese Academy of Medical Sciences and Peking Union Medical College, Beijing, China; 4 Department of Emergency, Fifth Medical Center of PLA General Hospital, Beijing, China; 5 Nursing Department, Fifth Medical Center of PLA General Hospital, Beijing, China

**Keywords:** Self-poisoning suicide, Mental health, Anxiety, Depression, Family members, Matched-pair analysis

## Abstract

**Background:**

The objective of this study was to examine the relationship of mental health status between self-poisoning suicide patients and their family members, and it also sought to identify potential patient’s risk and parental factors for the prediction of suicide attempt, anxiety, and depression.

**Methods:**

In this study, 151 poisoned patients were prospectively included, and they were matched 1:1 with 151 family members. We gathered information on patient’s and their matched family member’s demographics, lifestyle choices, mental health status, level of intimacy, and history of psychiatry disease. The relationship of patient’s and their family member’s mental health state was investigated using a correlation matrix. Multivariable analyses (multiple logistic regression) were conducted among patients and their matched family members, to identify potential risk factors for self-poisoning suicide, anxiety, and depression.

**Results:**

Of the total patients, 67.55% (102/151) attempted self-poisoning suicide. Poisoned patients had more severe anxiety and depression symptoms than their matched family members, and this difference was even more pronounced among patients with self-poisoning suicide. Generalized anxiety disorder-7 (GAD-7) score for family members was significantly and favorably correlated with patient’s GAD-7 score after eliminating non-suicide patients and their matched family members. The patient health questionnaire-9 (PHQ-9) score showed a similar pattern, and the family member’s PHQ-9 score was strongly and favorably associated with patient’s PHQ-9 and Beck hopelessness scale-20 (BHS-20) score. Multivariable analysis showed that married marital status (*P* = 0.038), quitting smoking (*P* = 0.003), sedentary time of 1 to 6 h (*P* = 0.013), and participation in a sports more than five times per week (*P* = 0.046) were all significantly associated with a lower risk of suicide by self-poisoning, while a more serious anxiety state (*P* = 0.001) was significantly associated with a higher risk of self-poisoning suicide. Multivariable analysis demonstrated that, specifically among self-poisoning suicide patients, married marital status (*P* = 0.011) and no history of psychiatry disease (*P* < 0.001) were protective factors for anxiety, while divorced or widowed marital status (*P* = 0.004), a sedentary time of 1 to 3 h (*P* = 0.022), and a higher monthly income (*P* = 0.027) were significant contributors to anxiety. The propensity of additional family-matched characteristics to predict patient’s suicidality, anxiety, and depression was also examined.

**Conclusions:**

Self-poisoning suicide patients have severe mental health issues. Patients who self-poison have a close connection to their family member’s mental health, particularly their levels of anxiety and depression. According to the findings, being married and adopting healthy lifestyle habits, such as quitting smoking and drinking, increasing their physical activity levels, and managing their idle time, are able to help patients with mental health concerns and even suicidal thoughts.

**Supplementary Information:**

The online version contains supplementary material available at 10.1186/s12888-023-04779-9.

## Background

Around the world, self-poisoning is a frequent method of ending one’s life, and poisoning can be caused by exposure to toxins chemicals, and drug overdoses [1]. It is a serious public health issue that contributes to global morbidity and mortality, particularly in low- and middle-income nations [1]. Self-poisoning is particularly prevalent in China, and a recent study pointed out that self-poisoning was responsible for roughly 60% of suicides [2]. In addition, both the prevalence and rate of suicide attempts using self-poisoning are dramatically rising among young people [3, 4].

Based on epidemiological and toxicological data, it was determined that national restrictions on harmful pesticides were an effective strategy for preventing suicide by pesticides intake [5, 6]. Even so, illicit drugs and medically prescribed drugs, such as psychotropic drugs, sedative-hypnotics, analgesics, and antidepressants [7], are still readily accessible and can be the most frequently misused drugs [8]. Identification of risk and protective factors is a crucial component of national suicide prevention since it helps to further understand the kind and type of preventive actions needed. Risk factors can occur on a variety of scales, including the individual, sociocultural, and situational, and numerous factors have been linked to increased self-poisoning, including borderline personality disorder or traits [9, 10], alcohol use disorder [9], depressive disorder [9], substance abuse [11, 12], asthma [11], fewer social connections [13], living in neighborhoods [13], being female [10, 14], and being young [13]. However, to the author’s best knowledge, there were few research that looked at the relationship between the mental health state of self-poisoning patients and their family members. It is important to stress that a thorough understanding of the risk factors for suicide behaviors in terms of patients and family members is necessary to carry out prevention activities, which highlights the need for a wider investigation.

In addition, during the Corona Virus Disease 2019 (COVID-19) pandemic, lockdowns and other public health measures had a significant impact on suicide [15]. Researchers noticed a decline in hospital admissions for self-poisoning suicide during the great pandemic, and, of note, this underlying trend actually reflected a decline in the number of patients receiving medical care for self-poisoning rather than a real decline in incidence. Thus, early professional healthcare seeking should be emphasized more, especially during the great pandemic. Identification of risk factors is an important step in guiding suicide prevention.

Therefore, the purpose of this study was to examine the relationship of mental health status between self-poisoning suicide patients and their family members, as well as to identify potential risk factors for predicting suicide attempt, anxiety, and depression using information from both the patients and their family members. In this study, it was expected that new risk factors for self-poisoning could be discovered from patients and their relatives. The study’s findings will be very helpful in directing the implementation of preventive interventions from the perspective of patients and their families.

## Patients and methods

### Patients

**Fig. 1:**
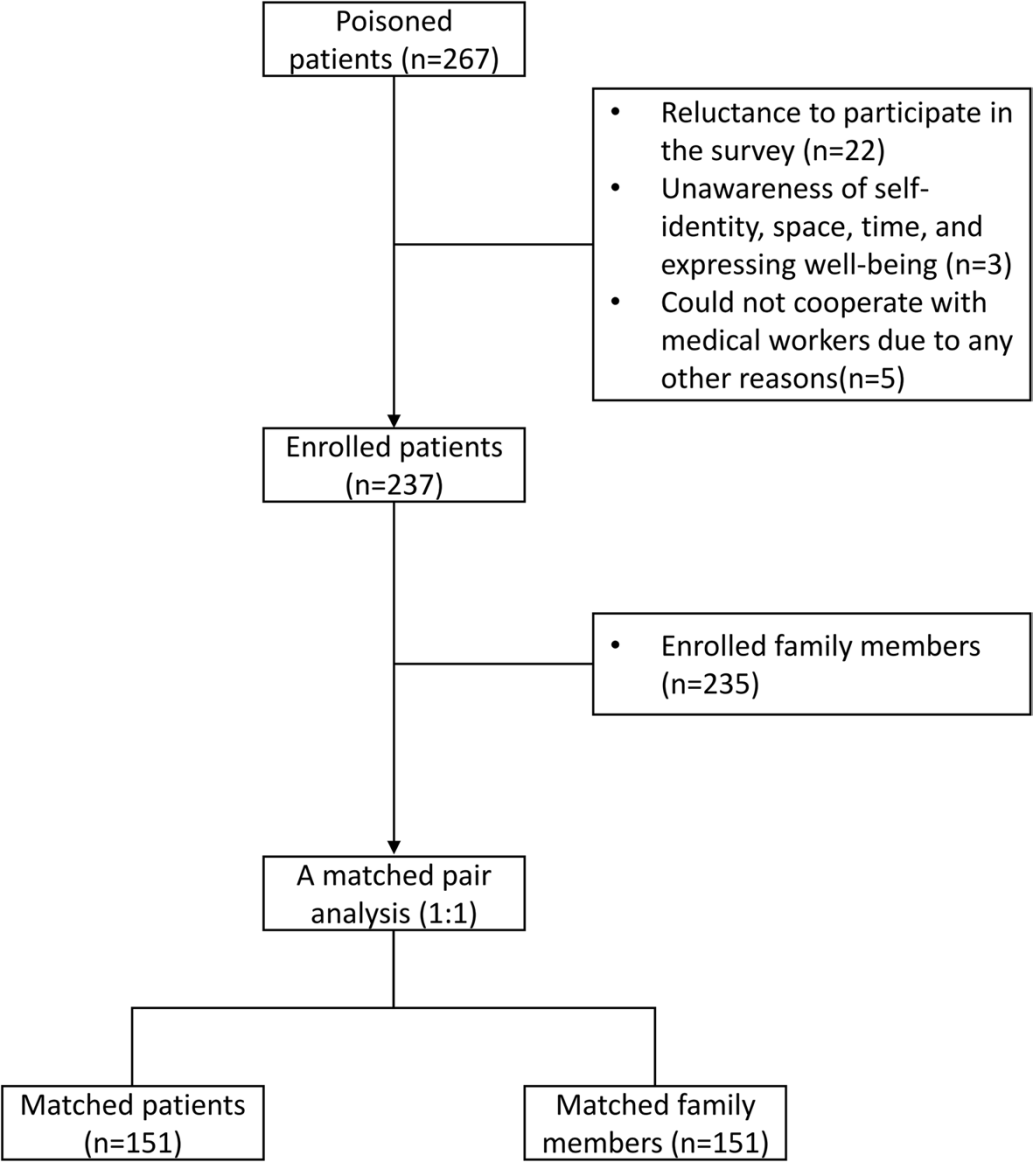
Patient’s diagram and study design

Between May 2021 and May 2022, this study prospectively enrolled 151 poisoned patients and 151 family members based on a matched rate of 1:1. We collected basic demographics, living habits, history of psychiatric diseases, and mental health status among patients and their counterpart family members. All included patients were admitted to our hospital for the treatment of poisoning. Patients were excluded for analysis if they (1) were unwilling to participate, (2) were unaware of self-identity, space, time, and expressing well-being, and (3) could not cooperate with medical workers or researchers for any other reasons. Family members were excluded if he or she (1) refused to take part in the study or (2) could not adequately articulate their well-being. The inclusive and exclusive criteria resulted in the collection of 237 patients and 236 family members. According to the matched rate of 1:1, 151 poisoned patients were matched with 151 family members in terms of patient’s admission ID. Figure 1 shows patient’s diagram. The study eliminated unmatched patients and family members from analysis. The Ethics Committee of the Fifth Medical Center of the Peoples Liberation Army (PLA) General Hospital approved the study protocol (No. KY-2021–12-34–1). All patients and family members provided written informed consent before the data were anonymously analyzed. The Helsinki Declaration was followed in this investigation.

### Characteristics of patients and their family members

All clinical traits among patients were categorized into the following types: (1) The patient’s basic demographic (gender, age, marital status, education level, and habitation geographic area), (2) their lifestyles (bland diet, greasy food, smoking, drinking, sedentary time, sport frequency per week, and monthly income), (3) their mental health status (anxiety, depression, self-esteem, hopelessness status, and social support status), and (4) their history of psychiatric diseases (history of depression and history of psychiatry disease). Anxiety and depression were assessed using generalized anxiety disorder-7 (GAD-7) [16] and patient health questionnaire-9 (PHQ-9) [16], respectively. Patient’s self-esteem was evaluated using self-esteem scale-10 (SES-10) [17], hopelessness status was measured using Beck hopelessness scale-20 (BHS-20) [18], and social support status was assessed using social support questionnaire-10 (SSQ-10) [19]. All the above scales, score ranges, explanations, Cronbach’s α, and relevant references are summarized in Supplementary table 1. All aspects of the patient’s lifestyle were self-reported, and a face-to-face interview was used to evaluate patient’s mental health. Poisoning patients were divided into two categories: self-poisoning and accidental poisoning. Self-poisoning suicide and method of self-poisoning, such as medicinal overdose, pesticide, or toxic chemicals, was initially identified using patient’s admission medical record, and verbally reconfirmed through patient’s self-report and their family members.

Regarding patient’s family members, this study collected their demographics including family relationship with patients, gender, age, living arrangement, and level of education, living habits (including sedentary time per day, sport frequency per week, drinking, smoking, and monthly income), intimacy score with patients, and mental health including anxiety and depression. Intimacy score was reported by family members and it ranges from 0 to 100 with higher scores indicating higher levels of intimacy between patients and their family members, and the score was adopted based on previous studies [20, 21]. The GAD-7 and PHQ-9 scales were used, respectively, to assess anxiety and depression.

### Statistical analysis

For categorical variables in the study, all data were summarized as the format of proportion, and for continuous variables, data were presented as mean and standard deviation (SD). Between non-suicide patients and suicide patients, as well as between their families, a comparison of characteristics was performed. The comparison of categorical variables was evaluated using the Chi-square test or continuous adjusted Chi-square test, while the comparison of continuous variables was examined using the *t* test or rank test. Correlation matrix was used to investigate the association of mental health status between patients and their family members, and specifically between suicide patients and their matched family members. In the correlation matrix, corresponding Pearson correlation coefficients, scatter diagrams, and histograms were all presented. To identify potential risk factors for self-poisoning suicide (no vs. yes), anxiety (none vs. mild vs. moderate vs. severe), and depression (none vs. mild vs. moderate vs. severe), multivariable analyses (multiple logistic regression) were performed in terms of patients and their family members, respectively. After adjusting for age and gender specifically among significant characteristics, this study performed a new analysis and verified their statistical significance. Finally, the study provided a summary of the risk factors for suicide, anxiety, and depression among patients and their families. Data were analyzed using R programming language (version 4.1.2). Statistical significance was established as a *P*-value of less than 0.05 with two-tailed sides.

## Results

### Characteristics of self-poisoning patients and their family members

To investigate the characteristics of suicide patients by self-poisoning, a comparison was performed between poisoned patients with and without suicide. When compared to poisoned patients who did not attempt suicide, self-poisoning patients were more likely to be female (66.7% vs. 42.9%, *P* = 0.009), younger (28.02 years old vs. 38.84 years old), and single (53.09% vs. 8.2%) (Table 1). Also, suicide patients did significantly less frequency of sports each week (*P* < 0.001) and had significantly lower monthly income (*P* = 0.013).

**Table 1:** A comparison of characteristics according to the presence of self-poisoning suicide among patients

**Characteristics**	**Overall**	**Suicide**		***P***
		**No**	**Yes**	
n	151	49	102	
Gender (%)				0.009
Male	62 (41.1)	28 (57.1)	34 (33.3)	
Female	89 (58.9)	21 (42.9)	68 (66.7)	
Age (mean (SD), years)	31.53 (14.72)	38.84 (12.33)	28.02 (14.53)	< 0.001
Marital status (%)				< 0.001
Single	59 (39.1)	4 (8.2)	55 (53.9)	
Dating	9 (6.0)	3 (6.1)	6 (5.9)	
Married	76 (50.3)	41 (83.7)	35 (34.3)	
Divorced or widowed	7 (4.6)	1 (2.0)	6 (5.9)	
Education level (%)				0.976
Primary	52 (34.4)	16 (32.7)	36 (35.3)	
High school	36 (23.8)	12 (24.5)	24 (23.5)	
University	58 (38.4)	19 (38.8)	39 (38.2)	
Graduate	5 (3.3)	2 (4.1)	3 (2.9)	
Habitation geographic area (%)				0.181
City	102 (67.5)	29 (59.2)	73 (71.6)	
Countryside	49 (32.5)	20 (40.8)	29 (28.4)	
Bland diet (%)				0.370
No	68 (45.0)	19 (38.8)	49 (48.0)	
Yes	83 (55.0)	30 (61.2)	53 (52.0)	
Greasy food (%)				0.073
No	129 (85.4)	46 (93.9)	83 (81.4)	
Yes	22 (14.6)	3 (6.1)	19 (18.6)	
Smoking (%)				0.424
Yes	45 (29.8)	12 (24.5)	33 (32.4)	
No	106 (70.2)	37 (75.5)	69 (67.6)	
Drinking (%)				0.540
Yes	24 (15.9)	6 (12.2)	18 (17.6)	
No	127 (84.1)	43 (87.8)	84 (82.4)	
Sedentary time (%, hours)				0.169
Less than 1	37 (24.5)	9 (18.4)	28 (27.5)	
1 ~ 3	61 (40.4)	25 (51.0)	36 (35.3)	
3 ~ 6	28 (18.5)	10 (20.4)	18 (17.6)	
Above 6	25 (16.6)	5 (10.2)	20 (19.6)	
Sport frequency per week (%)				< 0.001
0	33 (21.9)	8 (16.3)	25 (24.5)	
1 ~ 2	63 (41.7)	16 (32.7)	47 (46.1)	
3 ~ 5	35 (23.2)	10 (20.4)	25 (24.5)	
Above 5	20 (13.2)	15 (30.6)	5 (4.9)	
Monthly income (%, ￥)				0.013
Less than 3000	82 (54.3)	21 (42.9)	61 (59.8)	
3000 ~ 6000	52 (34.4)	19 (38.8)	33 (32.4)	
6000 ~ 9000	8 (5.3)	2 (4.1)	6 (5.9)	
Above 9000	9 (6.0)	7 (14.3)	2 (2.0)	
Severity of anxiety (GAD-7, %) ^a^				< 0.001
None	48 (31.8)	37 (75.5)	11 (10.8)	
Mild	33 (21.9)	9 (18.4)	24 (23.5)	
Moderate	38 (25.2)	1 (2.0)	37 (36.3)	
Severe	32 (21.2)	2 (4.1)	30 (29.4)	
Severity of depression (PHQ-9, %) ^a^				< 0.001
None	47 (31.1)	36 (73.5)	11 (10.8)	
Mild	17 (11.3)	5 (10.2)	12 (11.8)	
Moderate	24 (15.9)	4 (8.2)	20 (19.6)	
Severe	63 (41.7)	4 (8.2)	59 (57.8)	
SES-10 (mean (SD))	26.46 (6.16)	30.33 (5.08)	24.60 (5.77)	< 0.001
BHS-20 (mean (SD))	9.63 (4.29)	6.27 (3.17)	11.25 (3.80)	< 0.001
SSQ-10 (mean (SD))	36.21 (11.53)	41.96 (12.69)	33.44 (9.85)	< 0.001
History of depression (%)				< 0.001
Yes	56 (37.1)	5 (10.2)	51 (50.0)	
No	95 (62.9)	44 (89.8)	51 (50.0)	
History of psychiatry disease (%)				0.012
Yes	28 (18.5)	3 (6.1)	25 (24.5)	
No	123 (81.5)	46 (93.9)	77 (75.5)	

*SD* Standard deviation, *GAD-7* Generalized anxiety disorder-7, *PHQ-9* Patient health questionnaire-9, *SES-10* Self-esteem scale-10, *BHS-20* Beck hopelessness scale-20, *SSQ-10* Social support questionnaire-10

^a^none anxiety or depression indicates a GAD-7 or PHQ-9 score of 0 to 4, mild anxiety or depression indicates a score of 5 to 9, moderate anxiety or depression indicates a score of 10 to 14, and severe anxiety or depression indicates a score of 15 or above

Regarding mental health status, suicide patients had significant higher severity of anxiety (*P* < 0.001) and depression (*P* < 0.001), lower SES-10 (*P* < 0.001) and SSQ-10 (*P* < 0.001) scores, and a higher BHS-20 score (*P* < 0.001) than patients who did not attempt suicide. The findings indicated that suicide patients had severe mental health problems. What’s more, compared to patients without suicide, suicide patients had a significantly higher rate of history of depression (*P* < 0.001) and psychiatry disease (*P* = 0.012).

In terms of the characteristics of self-poisoning patient’s family members, suicide patient’s family members were more likely to be the patient’s parents (*P* = 0.002), have an education level of high school (*P* = 0.003), and have an introvert personality (*P* = 0.015) than non-suicide patient’s family members (Table 2). Nonetheless, the two groups did not differ significantly in terms of anxiety (*P* = 0.110) and depression (*P* = 0.190).

**Table 2:** A comparison of characteristics in terms of patient’s family members based on the presence of self-poisoning suicide among patients

**Characteristics**	**Overall**	**Suicide**		***P***
		**No**	**Yes**	
n	151	49	102	
Family relationship (%)				0.002
Parents	59 (39.1)	8 (16.3)	51 (50.0)	
Spouse	42 (27.8)	21 (42.9)	21 (20.6)	
Siblings	4 (2.6)	2 (4.1)	2 (2.0)	
Kids	12 (7.9)	4 (8.2)	8 (7.8)	
Others	34 (22.5)	14 (28.6)	20 (19.6)	
Gender (%)				0.396
Male	55 (36.4)	15 (30.6)	40 (39.2)	
Female	96 (63.6)	34 (69.4)	62 (60.8)	
Age (mean (SD), years)	42.15 (9.85)	42.49 (10.59)	41.99 (9.53)	0.772
Live together (%)				0.468
Yes	109 (72.2)	33 (67.3)	76 (74.5)	
No	42 (27.8)	16 (32.7)	26 (25.5)	
Education level (%)				0.003
Primary	60 (39.7)	23 (46.9)	37 (36.3)	
High school	37 (24.5)	3 (6.1)	34 (33.3)	
University	48 (31.8)	20 (40.8)	28 (27.5)	
Graduate	6 (4.0)	3 (6.1)	3 (2.9)	
Sedentary time (%, hours)				0.087
Less than 1	38 (25.2)	11 (22.4)	27 (26.5)	
1 ~ 3	60 (39.7)	14 (28.6)	46 (45.1)	
3 ~ 6	21 (13.9)	9 (18.4)	12 (11.8)	
Above 6	32 (21.2)	15 (30.6)	17 (16.7)	
Sport frequency per week (%)				0.134
0	17 (11.3)	9 (18.4)	8 (7.8)	
1 ~ 2	72 (47.7)	23 (46.9)	49 (48.0)	
3 ~ 5	28 (18.5)	10 (20.4)	18 (17.6)	
Above 5	34 (22.5)	7 (14.3)	27 (26.5)	
Drinking (%)				1.000
Yes	11 (7.3)	4 (8.2)	7 (6.9)	
No	140 (92.7)	45 (91.8)	95 (93.1)	
Smoking (%)				0.478
Yes	28 (18.5)	7 (14.3)	21 (20.6)	
No	123 (81.5)	42 (85.7)	81 (79.4)	
Monthly income (%, ￥)				0.107
Less than 3000	52 (34.4)	11 (22.4)	41 (40.2)	
3000 ~ 6000	59 (39.1)	20 (40.8)	39 (38.2)	
6000 ~ 9000	21 (13.9)	10 (20.4)	11 (10.8)	
Above 9000	19 (12.6)	8 (16.3)	11 (10.8)	
Personality (%)				0.015
Outgoing	68 (45.0)	21 (42.9)	47 (46.1)	
Middle	56 (37.1)	23 (46.9)	33 (32.4)	
Introvert	16 (10.6)	0 (0.0)	16 (15.7)	
Unclear	11 (7.3)	5 (10.2)	6 (5.9)	
Intimacy score (mean (SD))	88.13 (16.74)	88.20 (13.48)	88.09 (18.16)	0.968
Severity of anxiety (GAD-7, %) ^a^				0.110
None	99 (65.6)	29 (59.2)	70 (68.6)	
Mild	31 (20.5)	15 (30.6)	16 (15.7)	
Moderate	17 (11.3)	5 (10.2)	12 (11.8)	
Severe	4 (2.6)	0 (0.0)	4 (3.9)	
Severity of depression (PHQ-9, %) ^a^				0.190
None	95 (62.9)	27 (55.1)	68 (66.7)	
Mild	26 (17.2)	12 (24.5)	14 (13.7)	
Moderate	19 (12.6)	8 (16.3)	11 (10.8)	
Severe	11 (7.3)	2 (4.1)	9 (8.8)	

*SD* Standard deviation, *GAD-7* Generalized anxiety disorder-7, *PHQ-9* Patient health questionnaire-9

^a^none anxiety or depression indicates a GAD-7 or PHQ-9 score of 0 to 4, mild anxiety or depression indicates a score of 5 to 9, moderate anxiety or depression indicates a score of 10 to 14, and severe anxiety or depression indicates a score of 15 or above

### Comparison of mental health status between patients and their family members

**Fig. 2:**
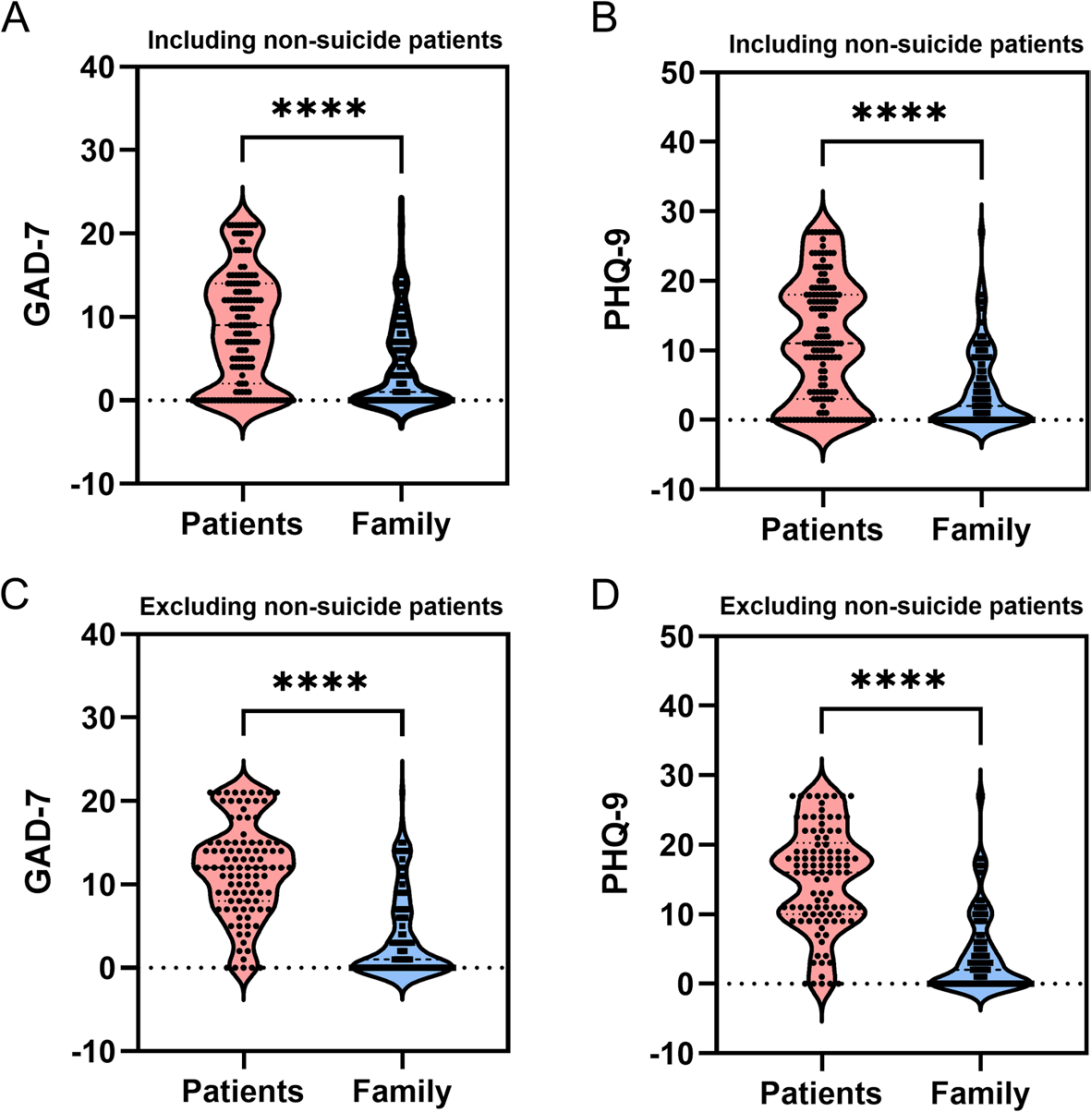
Subgroup analysis of anxiety and depression between patients and their matched family members. **A** A comparison of GAD-7 scores among all enrolled patients. **B** A comparison of PHQ-9 scores among all enrolled patients. **C** A comparison of GAD-7 scores specifically among self-poisoning patients. **D** A comparison of PHQ-9 scores specifically among self-poisoning patients

A matched-pair analysis showed that poisoned patients had significant higher GAD-7 (*P* < 0.001, Fig. 2A) and PHQ-9 (*P* < 0.001, Fig. 2B) scores than their matched family members. After eliminating poisoned patients who did not attempt suicide, self-poisoning suicide patients had even significant higher GAD-7 (*P* < 0.001, Fig. 2C) and PHQ-9 (*P* < 0.001, Fig. 2D) scores than their matched family members. To clarify, when non-suicide patients were not included for analysis, subgroup analysis of suicide patients revealed that the difference in GAD-7 increased from 4.97 (95%CI: 3.69–6.26) to 7.91 (95%CI: 6.55–9.28) and the difference in PHQ-9 increased from 6.91 (95%CI: 5.27–8.56) to 10.71 (95%CI: 9.04–12.38). Therefore, poisoned patients experienced more severe anxiety and depression than their matched family members, and this pattern was especially pronounced among patients who attempted suicide and their matched family members.

**Fig. 3:**
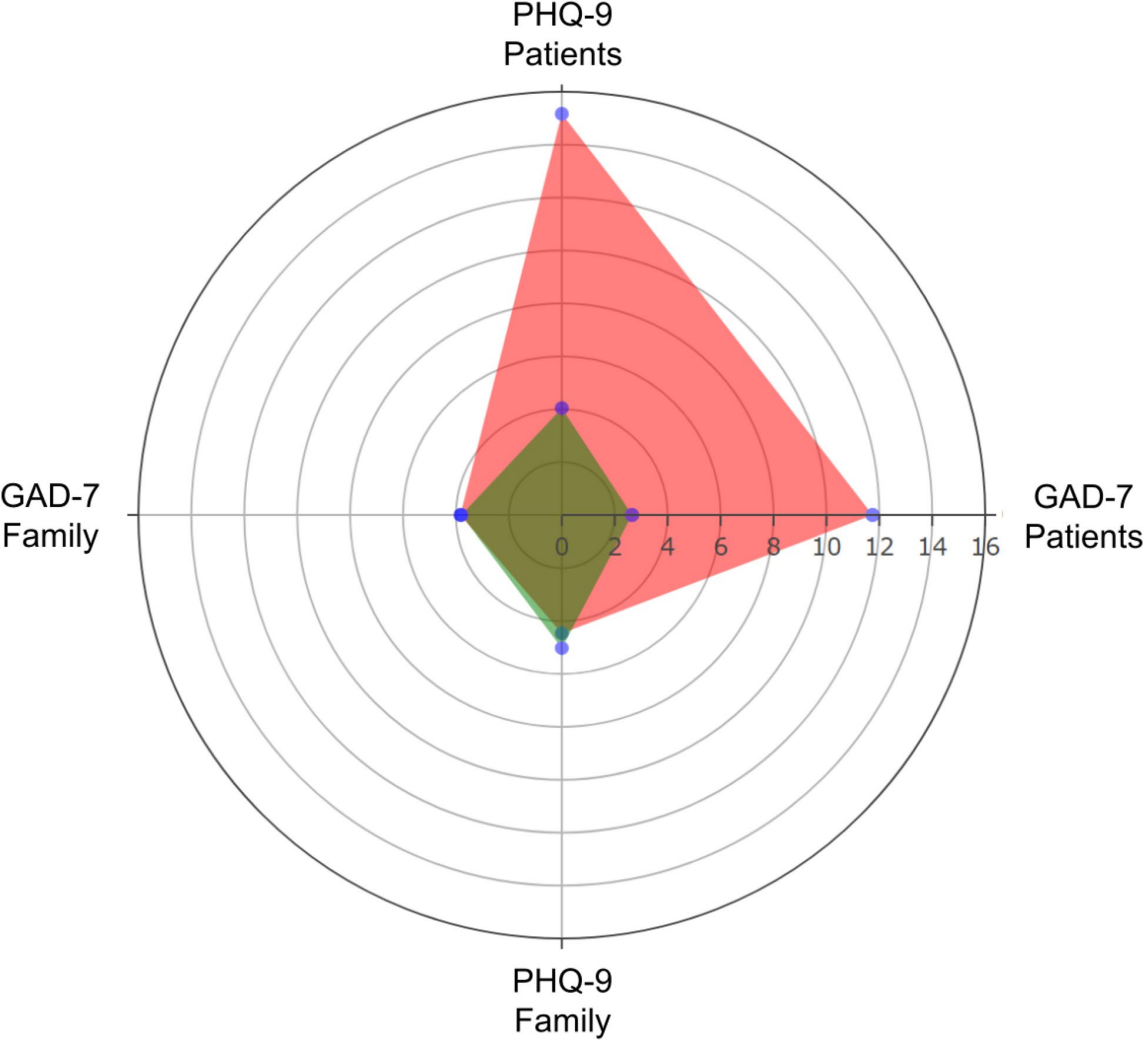
Radar plots of anxiety and depression according to the presence of self-poisoning suicide among patients and their matched family members. Red indicates self-poisoning suicide patients or their matched family members; Green indicates patients without self-poisoning suicide or their matched family members. GAD-7, Generalized anxiety disorder-7; PHQ-9, Patient health questionnaire-9

**Fig. 4:**
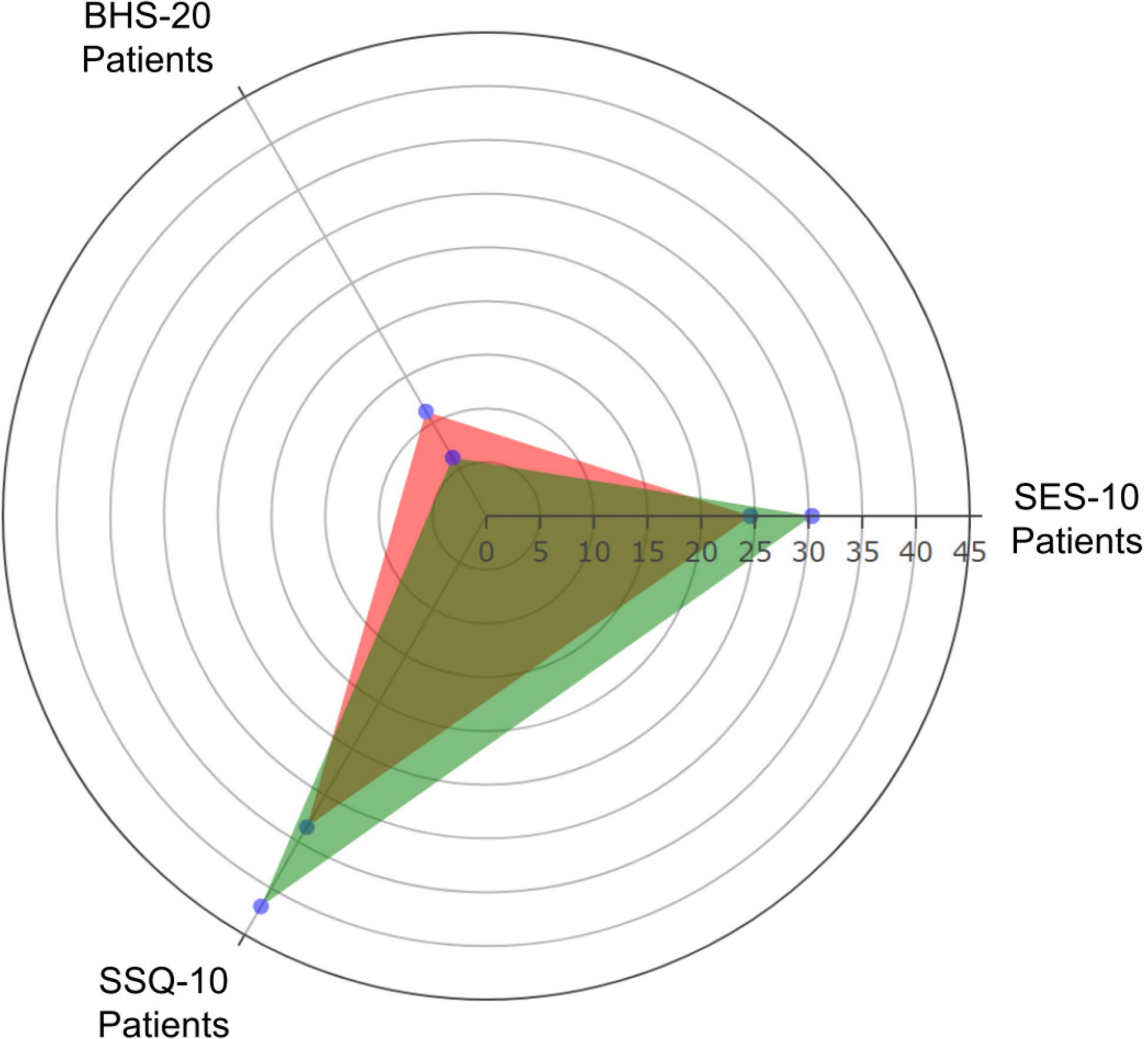
Radar plots of hopelessness, self-esteem, and social support status according to the presence of self-poisoning suicide among patients. Red indicates self-poisoning suicide patients; Green indicates patients without self-poisoning suicide. SES-10, Self-esteem scale-10; BHS-20, Beck hopelessness scale-20; SSQ-10, Social support questionnaire-10

In addition, self-poisoning suicide patients had significant higher GAD-7 (*P* < 0.001) and PHQ-9 (*P* < 0.001) scores compared to patients without suicide (Fig. 3). However, both GAD-7 and PHQ-9 scores were very similar between suicide patient’s family members and non-suicide patient’s family members. As anticipated, suicide patients performed worse on the BHS-20 (*P* < 0.001), lower SES-10 (*P* < 0.001) and SSQ-10 (*P* < 0.001) scores than their counterparts (Fig. 4). The data above showed that, in contrast to suicide patients, their family members did not experience serious mental health issues.

### Association of mental health status between patients and their family members

**Fig. 5:**
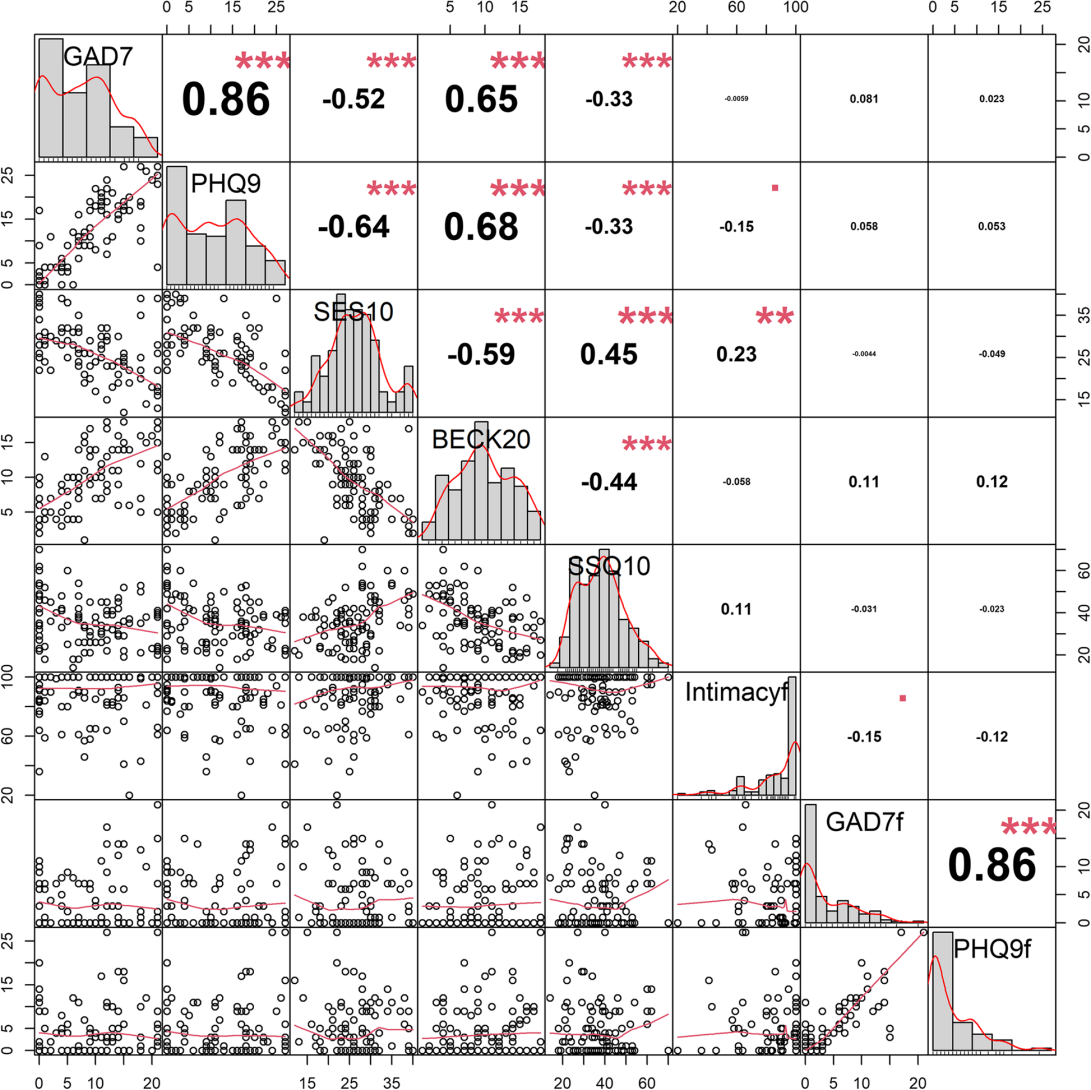
Correlation matrix for scales including patient’s anxiety, depression, self-esteem, hopelessness, and social support status, family member’s anxiety and depression, and intimacy score between patients and family members among all enrolled patients and their matched family members. Lower left panels depict the scatted diagrams between scales, Upper right panels depict the correlation coefficients between scales and significance is given (* indicates *P*<0.05, ** indicates *P*<0.01, *** indicates *P*<0.001), and diagonal panels depict histograms of scales. GAD-7, Generalized anxiety disorder-7; PHQ-9, Patient health questionnaire-9; SES-10, Self-esteem scale-10; BECK-20 indicates BHS-20, Beck hopelessness scale-20; SSQ-10, Social support questionnaire-10; “Intimacyf” indicates intimacy score between patients and their family members and it was reported by family members; GAD-7f, Generalized anxiety disorder-7 for family members; PHQ-9f, Patient health questionnaire-9 for family members

**Fig. 6:**
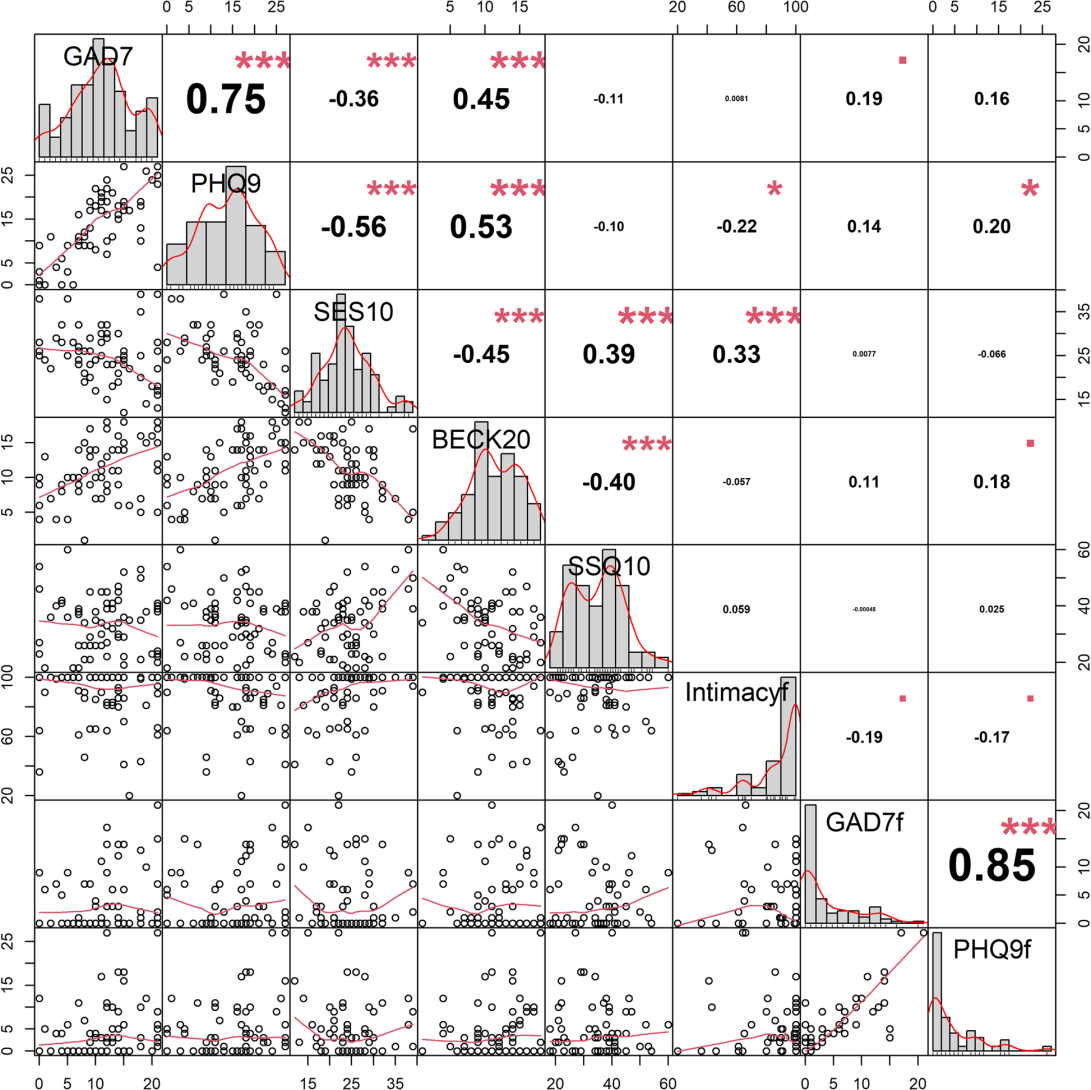
Correlation matrix for scales including patient’s anxiety, depression, self-esteem, hopelessness, and social support status, family member’s anxiety and depression, and intimacy score between patients and family members specifically among self-poisoning patients and their matched family members. Lower left panels depict the scatted diagrams between scales, Upper right panels depict the correlation coefficients between scales and significance is given (* indicates *P*<0.05, ** indicates *P*<0.01, *** indicates *P*<0.001), and diagonal panels depict histograms of scales. GAD-7, Generalized anxiety disorder-7; PHQ-9, Patient health questionnaire-9; SES-10, Self-esteem scale-10; BECK-20 indicates BHS-20, Beck hopelessness scale-20; SSQ-10, Social support questionnaire-10; “Intimacyf” indicates intimacy score between patients and their family members and it was reported by family members; GAD-7f, Generalized anxiety disorder-7 for family members; PHQ-9f, Patient health questionnaire-9 for family members

Correlation matrix was used to visualize the association of mental health between patients and their matched family members. Among all poisoned patients, Fig. 5 demonstrated that patient’s GAD-7, PHQ-9, SES-10, BHS-20, and SSQ-10 scores were significantly interrelated with each other. Also, among the patient’s family members, there was a strong correlation between GAD-7 and PHQ-9 scores. Family members reported intimacy score with patients were statistically relevant to patient’s PHQ-9 and SES-10 scores. Nonetheless, family member’s GAD-7 and PHQ-9 scores were not significantly associated with patient’s mental health. However, when non-suicide patients and their family members were taken out, the GAD-7 score of the family member was significantly and favorably correlated with the patient’s GAD-7 score and negatively correlated with the intimacy score (Fig. 6). The PHQ-9 score showed a similar pattern; family member’s PHQ-9 score was significantly and favorably related to patient’s PHQ-9 and BHS-20 scores and adversely related to the intimacy score. Pearson correlation coefficients, scatter diagrams, and histograms were all shown.

### Risk factors associated with self-poisoning suicide

**Fig. 7:**
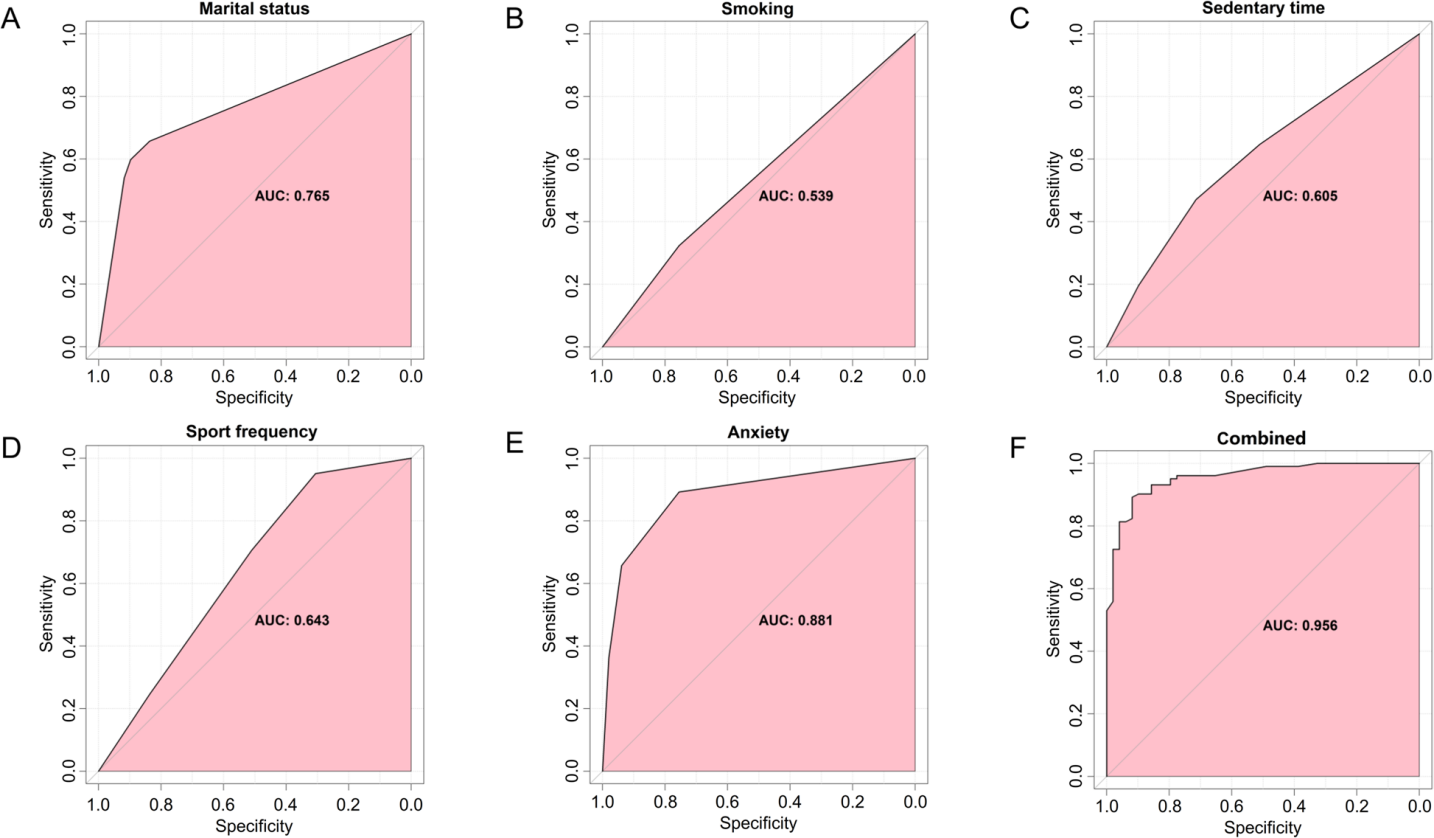
The area under the curve of significant risk factors for predicting self-poisoning suicide. **A** Marital status. **B** Smoking. **C** Sedentary time. **D** Sport frequency. **E** Anxiety. **F** All the five features combined together

Multivariable analysis showed that married marital status (*P* = 0.038), not smoking (*P* = 0.003), sedentary time of 1 to 6 h (*P* = 0.013), and participation in sports more than five times per week (*P* = 0.046) were significantly associated with a lower risk of suicide by self-poisoning, while a severer anxious state (*P* = 0.001) was a significant contributor to self-poisoning suicide (Table 3). In the predictive evaluation of risk factors for self-poisoning suicide, the area under the curve (AUC) values for the five variables were 0.765 (95% CI: 0.699–0.832, Fig. 7A), 0.539 (95% CI: 0.463–0.615, Fig. 7B), 0.605 (95% CI: 0.515–0.69, Fig. 7C), 0.643 (95% CI: 0.547–0.739, Fig. 7D), and 0.881 (95% CI: 0.827–0.936, Fig. 7E), respectively, and when the five variables were combined, the AUC value was 0.956 (95% CI: 0.926–0.986, Fig. 7F). After adjusting for age and gender among the above significant characteristics, married marital status nearly attained significance (*P* = 0.062), whereas other five significant variables persisted in significance (Supplementary table 2).

**Table 3:** Multivariable analysis of characteristics for predicting self-poisoning suicide among patients (*n* = 151)

**Characteristics**	**OR**	**95% CI**		***P***
		**LL**	**UL**	
Intercept	1.64	1.15	2.32	0.007
Gender				
Male	Ref			
Female	1.14	1.00	1.31	0.055
Age	1.00	0.99	1.01	0.792
Marital status				
Single	Ref			
Dating	0.88	0.68	1.13	0.320
Married	0.80	0.65	0.98	0.038
Divorced or widowed	0.73	0.52	1.03	0.074
Education level				
Primary	Ref			
High school	0.93	0.79	1.10	0.415
University	0.91	0.78	1.06	0.220
Graduate	1.33	0.93	1.91	0.117
Habitation geographic area				
City	Ref			
Countryside	1.12	0.98	1.28	0.099
Bland diet				
No	Ref			
Yes	1.06	0.93	1.21	0.387
Greasy food				
No	Ref			
Yes	1.07	0.89	1.28	0.468
Smoking				
Yes	Ref			
No	0.78	0.66	0.91	0.003
Drinking				
Yes	Ref			
No	0.97	0.81	1.17	0.771
Sedentary time (hours)				
Less than 1	Ref			
1 ~ 3	0.82	0.71	0.96	0.013
3 ~ 6	0.81	0.67	0.98	0.035
Above 6	0.90	0.74	1.09	0.268
Sport frequency per week				
0	Ref			
1 ~ 2	1.24	1.05	1.45	0.011
3 ~ 5	1.04	0.87	1.25	0.637
Above 5	0.81	0.67	0.99	0.046
Monthly income (￥)				
Less than 3000	Ref			
3000 ~ 6000	0.97	0.84	1.12	0.720
6000 ~ 9000	1.08	0.78	1.48	0.644
Above 9000	0.82	0.63	1.07	0.138
Severity of anxiety (GAD-7) ^a^				
None	Ref			
Mild	1.33	1.07	1.66	0.012
Moderate	1.75	1.35	2.27	0.000
Severe	1.58	1.21	2.06	0.001
Severity of depression (PHQ-9) ^a^				
None	Ref			
Mild	1.20	0.95	1.52	0.125
Moderate	1.15	0.91	1.46	0.229
Severe	1.14	0.88	1.48	0.307
History of depression				
Yes	Ref			
No	1.04	0.89	1.22	0.579
History of psychiatry disease				
Yes	Ref			
No	1.11	0.91	1.35	0.300

*OR* Odds ratio, *CI* Confident interval, *LL* Lower limit, *UL* Upper limit, *GAD-7* Generalized anxiety disorder-7, *PHQ-9* Patient health questionnaire-9

^a^none anxiety or depression indicates a GAD-7 or PHQ-9 score of 0 to 4, mild anxiety or depression indicates a score of 5 to 9, moderate anxiety or depression indicates a score of 10 to 14, and severe anxiety or depression indicates a score of 15 or above

Regarding characteristics in terms of the matched family members, this study found that an education level of high school (*P* = 0.004) and an introvert personality (*P* = 0.023) were significantly associated with a greater rate of suicide (Table 4), while spouse (*P* < 0.001) and female (*P* = 0.047) were significant protective variables. After adjusting for age and gender among significant characteristics, the four variables were still significant (Supplementary table 3).

**Table 4:** Multivariable analysis of characteristics in terms of matched family members for predicting patient’s self-poisoning suicide (*n* = 151)

**Characteristics**	**OR**	**95% CI**		***P***
		**LL**	**UL**	
Intercept	5.95	2.50	14.18	0.000
Family relationship				
Parents	Ref			
Spouse	0.64	0.51	0.81	0.000
Siblings	0.79	0.47	1.32	0.377
Kids	0.82	0.62	1.09	0.167
Others	0.65	0.42	1.01	0.060
Gender				
Male	Ref			
Female	0.78	0.61	0.99	0.047
Age	0.99	0.98	1.00	0.055
Live together				
Yes	Ref			
No	0.91	0.71	1.16	0.442
Education level				
Primary	Ref			
High school	1.47	1.14	1.90	0.004
University	1.10	0.87	1.39	0.449
Graduate	1.03	0.67	1.59	0.890
Sedentary time (hours)				
Less than 1	Ref			
1 ~ 3	1.12	0.89	1.41	0.339
3 ~ 6	1.06	0.76	1.48	0.722
Above 6	0.83	0.63	1.10	0.206
Sport frequency per week				
0	Ref			
1 ~ 2	0.83	0.62	1.11	0.217
3 ~ 5	0.97	0.71	1.32	0.825
Above 5	0.84	0.60	1.17	0.302
Drinking				
Yes	Ref			
No	1.16	0.79	1.71	0.444
Smoking				
Yes	Ref			
No	0.93	0.70	1.24	0.623
Monthly income (￥)				
Less than 3000	Ref			
3000 ~ 6000	0.86	0.68	1.07	0.184
6000 ~ 9000	0.81	0.58	1.15	0.242
Above 9000	0.78	0.59	1.04	0.093
Character				
Outgoing	Ref			
Middle	0.95	0.79	1.14	0.571
Introvert	1.41	1.05	1.88	0.023
Unclear	0.80	0.55	1.17	0.258
Intimacy score	1.00	0.99	1.00	0.486
Severity of anxiety (GAD-7) ^a^				
None	Ref			
Mild	0.75	0.54	1.04	0.090
Moderate	0.82	0.51	1.32	0.418
Severe	1.31	0.57	2.98	0.524
Severity of depression (PHQ-9) ^a^				
None	Ref			
Mild	1.04	0.72	1.49	0.841
Moderate	1.12	0.78	1.62	0.537
Severe	1.00	0.58	1.74	0.989

*OR* Odds ratio, *CI* Confident interval, *LL* Lower limit, *UL* Upper limit, *GAD-7* Generalized anxiety disorder-7, *PHQ-9* Patient health questionnaire-9

^a^none anxiety or depression indicates a GAD-7 or PHQ-9 score of 0 to 4, mild anxiety or depression indicates a score of 5 to 9, moderate anxiety or depression indicates a score of 10 to 14, and severe anxiety or depression indicates a score of 15 or above

### Risk factors associated with anxiety and depression among self-poisoning suicide patients

To investigate risk factors related to anxiety and depression specifically among self-poisoning suicide patients, non-suicide patients were excluded for analysis. Multivariable analysis showed that married marital status (*P* = 0.011) and the absence of psychiatry disease history (*P* < 0.001) were protective factors against anxiety, while divorced or widowed marital status (*P* = 0.002), a sedentary time of 1 to 3 h (*P* = 0.022), and a higher monthly income (*P* = 0.027) were significant contributors to more anxiety specifically among suicide patients by self-poisoning (Table 5). Sedentary time lost significance after correcting for age and gender, but other three variables remained significance (Supplementary table 4). Higher age (*P* = 0.010) and monthly income (*P* = 0.008) were significant risk factors for depression, whereas married marital status (*P* < 0.001), abstinence from alcohol (*P* = 0.007), and no history of psychiatry illness (*P* < 0.001) were significant protective factors for depression. After adjusting for age and gender among significant characteristics, drinking did not achieve significance, but the other four variables still did (Supplementary table 5).

**Table 5:** Multivariable analysis of characteristics for predicting patient’s anxiety and depression specifically among patients with self-poisoning suicide (*n* = 102)

**Characteristics**	**Anxiety**				**Depression**			
	**OR**	**95% CI**		***P***	**OR**	**95% CI**		***P***
		**LL**	**UL**			**LL**	**UL**	
Intercept	34.08	14.69	79.04	0.000	31.93	13.49	75.57	0.000
Gender								
Male	Ref				Ref			
Female	1.23	0.81	1.87	0.338	1.38	0.90	2.12	0.148
Age	1.01	0.99	1.03	0.358	1.03	1.01	1.06	0.010
Marital status								
Single	Ref				Ref			
Dating	0.62	0.29	1.30	0.208	1.06	0.49	2.27	0.891
Married	0.37	0.17	0.78	0.011	0.21	0.10	0.45	0.000
Divorced or widowed	4.10	1.72	9.73	0.002	1.78	0.73	4.31	0.208
Education level								
Primary	Ref				Ref			
High school	1.07	0.63	1.83	0.796	1.38	0.80	2.37	0.254
University	0.84	0.53	1.33	0.465	0.96	0.60	1.52	0.850
Graduate	1.43	0.40	5.13	0.580	0.84	0.23	3.10	0.798
Habitation geographic area								
City	Ref				Ref			
Countryside	0.74	0.48	1.13	0.163	0.82	0.53	1.27	0.369
Bland diet								
No	Ref				Ref			
Yes	0.86	0.57	1.31	0.493	0.66	0.43	1.01	0.059
Greasy food								
No	Ref				Ref			
Yes	1.34	0.83	2.19	0.237	1.43	0.87	2.36	0.161
Smoking								
Yes	Ref				Ref			
No	1.10	0.66	1.83	0.710	1.60	0.95	2.68	0.080
Drinking								
Yes	Ref				Ref			
No	0.81	0.43	1.50	0.497	0.40	0.21	0.76	0.007
Sedentary time (hours)								
Less than 1	Ref				Ref			
1 ~ 3	1.76	1.09	2.84	0.022	1.47	0.90	2.39	0.128
3 ~ 6	1.01	0.55	1.86	0.966	0.97	0.52	1.81	0.927
Above 6	1.38	0.74	2.57	0.313	0.98	0.52	1.85	0.944
Sport frequency per week								
0	Ref				Ref			
1 ~ 2	0.79	0.47	1.34	0.383	1.46	0.85	2.51	0.171
3 ~ 5	0.91	0.51	1.63	0.752	1.73	0.96	3.14	0.074
Above 5	1.00	0.39	2.57	1.000	1.75	0.67	4.61	0.259
Monthly income (￥)								
Less than 3000	Ref				Ref			
3000 ~ 6000	0.79	0.48	1.30	0.347	1.18	0.71	1.98	0.519
6000 ~ 9000	4.35	1.82	10.41	0.001	6.83	2.80	16.68	0.000
Above 9000	5.44	1.24	23.78	0.027	8.07	1.78	36.55	0.008
History of depression								
Yes	Ref				Ref			
No	1.03	0.67	1.58	0.894	0.83	0.53	1.28	0.399
History of psychiatry disease								
Yes	Ref				Ref			
No	0.36	0.21	0.61	0.000	0.34	0.20	0.59	0.000

*OR* Odds ratio, *CI* Confident interval, *LL* Lower limit, *UL* Upper limit

Regarding the variables in terms of their family members, an education level of high school (*P* = 0.040), relative higher monthly income (*P* = 0.035), and higher severity of anxiety (*P* = 0.011) were significant risk factors for patient’s anxiety (Table 6), whereas middle (*P* = 0.027) or unclear (*P* = 0.07) personality were protective. Age and gender adjustments among the above significant characteristics revealed that the all four variables were significant (Supplementary table 6). A sport frequency above five per week (*P* = 0.023) and a higher severity of depression (*P* = 0.013) among families were risk factors for patient’s depression, whereas middle personality (*P* = 0.030) and mild anxiety (*P* = 0.039) were protective factors. After adjusting for age and gender among significant characteristics, the four variables were still significant (Supplementary table 7).

**Table 6:** Multivariable analysis of characteristics in terms of family members for predicting patient’s anxiety and depression specifically among self-poisoning patients (*n* = 102)

**Characteristics**	**Anxiety**				**Depression**			
	**OR**	**95% CI**		***P***	**OR**	**95% CI**		***P***
		**LL**	**UL**			**LL**	**UL**	
Intercept	22.26	1.91	259.01	0.016	3.33	0.28	39.01	0.341
Family relationship								
Parents	Ref				Ref			
Spouse	1.23	0.57	2.67	0.596	0.93	0.43	2.02	0.858
Siblings	1.21	0.27	5.45	0.806	1.26	0.28	5.71	0.763
Kids	0.77	0.34	1.73	0.529	0.69	0.31	1.55	0.373
Others	1.25	0.64	2.43	0.523	1.27	0.65	2.49	0.484
Gender								
Male	Ref				Ref			
Female	1.71	0.92	3.16	0.094	1.48	0.80	2.75	0.215
Age	0.98	0.96	1.01	0.220	1.01	0.98	1.04	0.476
Live together								
Yes	Ref				Ref			
No	1.46	0.74	2.89	0.281	0.77	0.39	1.53	0.464
Education level								
Primary	Ref				Ref			
High school	2.27	1.05	4.90	0.040	0.95	0.44	2.05	0.895
University	1.08	0.49	2.40	0.850	0.59	0.26	1.31	0.196
Graduate	1.14	0.26	5.06	0.864	3.03	0.68	13.53	0.150
Sedentary time (hours)								
Less than 1	Ref				Ref			
1 ~ 3	0.97	0.53	1.77	0.909	1.44	0.79	2.65	0.241
3 ~ 6	1.09	0.44	2.74	0.850	1.34	0.53	3.36	0.538
Above 6	0.48	0.21	1.09	0.085	0.61	0.27	1.40	0.251
Sport frequency per week								
0	Ref				Ref			
1 ~ 2	1.59	0.59	4.27	0.362	1.53	0.57	4.12	0.403
3 ~ 5	1.13	0.41	3.12	0.822	1.76	0.63	4.89	0.285
Above 5	1.75	0.68	4.51	0.252	3.07	1.19	7.95	0.023
Drinking								
Yes	Ref				Ref			
No	0.87	0.34	2.24	0.776	2.16	0.84	5.59	0.115
Smoking								
Yes	Ref				Ref			
No	0.87	0.40	1.92	0.739	1.05	0.48	2.31	0.897
Monthly income (￥)								
Less than 3000	Ref				Ref			
3000 ~ 6000	1.03	0.58	1.83	0.910	0.85	0.48	1.51	0.592
6000 ~ 9000	2.01	0.79	5.13	0.149	1.48	0.58	3.79	0.415
Above 9000	2.29	1.08	4.88	0.035	1.84	0.86	3.93	0.118
Character								
Outgoing	Ref				Ref			
Middle	0.57	0.35	0.93	0.027	0.58	0.36	0.94	0.030
Introvert	1.44	0.72	2.88	0.306	1.67	0.83	3.36	0.151
Unclear	0.23	0.08	0.65	0.007	0.50	0.18	1.40	0.190
Intimacy score	1.00	0.98	1.01	0.676	1.00	0.99	1.02	0.755
Severity of anxiety (GAD-7) ^a^								
None	Ref				Ref			
Mild	0.37	0.16	0.85	0.022	0.40	0.17	0.94	0.039
Moderate	1.97	0.58	6.72	0.282	0.35	0.10	1.21	0.101
Severe	6.07	1.57	23.40	0.011	0.76	0.20	2.95	0.697
Severity of depression (PHQ-9) ^a^								
None	Ref				Ref			
Mild	1.22	0.52	2.86	0.642	1.54	0.66	3.62	0.321
Moderate	0.92	0.38	2.24	0.862	1.08	0.44	2.63	0.865
Severe	0.70	0.16	2.96	0.626	6.55	1.54	27.97	0.013

*OR* Odds ratio, *CI* Confident interval, *LL* Lower limit, *UL* Upper limit, *GAD-7* Generalized anxiety disorder-7, *PHQ-9* Patient health questionnaire-9

^a^none anxiety or depression indicates a GAD-7 or PHQ-9 score of 0 to 4, mild anxiety or depression indicates a score of 5 to 9, moderate anxiety or depression indicates a score of 10 to 14, and severe anxiety or depression indicates a score of 15 or above

### An overview of all risk factors

**Fig. 8:**
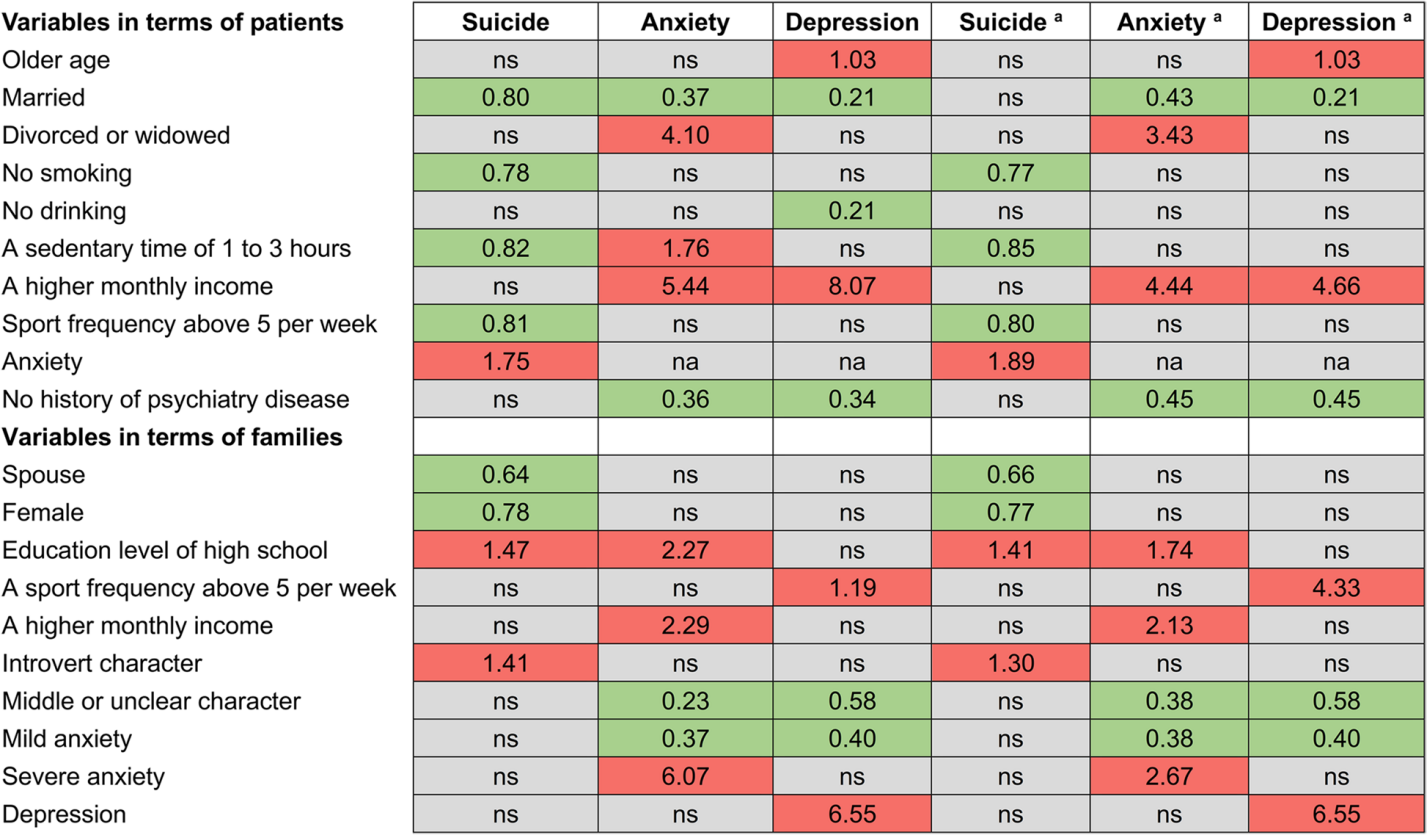
An overview of risk factors for suicide, anxiety, and depression based on the features in terms of patients or their family members. The summary of risk factors for anxiety and depression were specifically among self-poisoning patients. Red indicates risk factors; Green indicates protective factors. Odds ratio is summarized in the boxes. ^a^ indicates the results after adjusting for age and gender among significant characteristics

An overview of all risk factors for suicide, anxiety, and depression based on the features in terms of patients or their family members is shown in Fig. 8. The summary of risk factors for anxiety and depression were specifically among self-poisoning patients. It provided a thorough analysis of risk and parental factors. In detail, married marital status was a protective factor for suicide, anxiety, and depression among patients, and it could explain that spouse was a protective factor for preventing patient’s suicide. Regarding lifestyles, quitting smoking, giving up drinking, getting more exercise, and managing appropriate sedentary time were beneficial to reduce poor mental health and even suicide behavior. In addition, no prior history of psychiatry disease was linked to lower levels of anxiety and depression. Regarding the clinical characteristics of family members, sport frequency and monthly income were associated with depression and anxiety, respectively. An introvert personality among family members might have contributed to patient’s suicide. Of note, family member’s education level might also have a significant impact on the risk of suicide and anxiety, as this study showed that an education level of high school was associated with more patient’s suicide and anxiety. The overview of all risk factors also demonstrated that family member’s anxiety and depression were closely associated with patient’s anxiety and depression. In addition, the majority of factors still had the same importance after being adjusted for age and gender specifically among significant characteristics.

## Discussion

Suicide has developed into a serious global public health problem linked to significant psychological disability, mortality, and impairment [22]. This study showed that compared to non-suicide patients who were admitted to the hospital due to poisoning, self-poisoning suicide patients experienced more serious mental health issues, such as anxiety, depression, hopelessness, and a lack of social support. The findings highlighted the need for more mental health care for people who were prone to self-poisoning. Also, among self-poisoning patients in particular, family members’ mental health state was substantially correlated with patients’ psychiatric status, despite the fact that they scored statistically significantly lower on anxiety and depression than patients did. It suggested that not only self-poisoning patients but also their matched family members should seek professional assistance and access to mental healthcare. Hence, an emphasis on modifiable aspects is now necessary to enable the value of the evidence in suicide prevention.

In the present study, multivariable analysis showed that married marital status, sedentary time of 1 to 6 h, and participation in sports more than five times per week were significantly associated with a lower rate of self-poisoning suicide, while current smoking and anxiety were significant risk factors. Previous studies have shown that divorce was a risk factor for self-poisoning [23], and anxiety was one of most relevant risk factors for suicide [24]. In addition, anxiety disorders raised the chance of subsequent episodes of purposeful self-harm among adolescents and young adults [25]. A meta-analysis showed that depressive and anxious symptoms and comorbid substance use increased the risk of suicide attempts [26]. Another meta-analysis also demonstrated that anxiety increased suicide risk [27]. Interestingly, in this study, we further identified risk factors for self-poisoning based on variables from patient’s matched family members. Family member’s identity, gender, education level, and personality had an impact on patient’s self-poisoning rates. Spouse was severed as a protective factor which was confirmed by the result that married marital status was associated with a lower risk of self-poisoning suicide. A larger monthly income for family members was associated with a tendency for a reduction in the probability of self-poisoning suicide. Yet, patients with higher monthly incomes can experience more worry and depression as opposed to a reduction in the probability of self-poisoning suicide. To earn a high salary, a patient might need to work more and take on more tasks, which could lead to anxiety and sadness owing to the strain of the labor. Patients were more likely to receive financial assistance from their family members if they made a greater monthly income, which may have lessened the patient’s suicidal thoughts. In addition, high school education and an introverted personality trait among family members were additional risk factors for self-poisoning among patients. Previous study also showed that higher introversion increased the risk of suicide ideation [28], and our present study demonstrated that introvert personality among family members also increased the risk of suicide attempts. Family members with introvert personality might not have effective communications with patients, which might possibly affect the mental health of patients. Family members living together with patients might be beneficial to curb anxiety, and this could be explained that such accompany was a protective factor for anxious status. Previous study had already showed that female was vulnerable to self-poisoning suicide [10, 14], and our study also depicted a similar trend. But, interestingly, having female family members helped to prevent self-poisoning suicide for patients. The above-mentioned findings demonstrated a connection of psychiatric health between self-poisoning patients and their family members.

In addition, specifically among self-poisoning suicide patients, multivariable analysis demonstrated that married marital status and no history of psychiatry disease were protective factors for preventing anxiety and depression, while older age, divorced or widowed marital status, a sedentary time of 1 to 3 h, drinking, and a higher monthly income were significant contributors to anxiety or depression. As shown in the above, anxiety was one of main contributors to self-poisoning [25–27]. Our study further depicted concise suggestions for patients to deal with anxiety, such as finding a partner, treating previous psychiatry disease, and cultivating health lifestyle. Among the parental traits, a meta-analysis revealed less warmth, more interparental conflict, over-involvement, and aversiveness were linked to higher rates of depression and anxiety [29], indicating that a peaceful home environment was helpful in preventing anxiety and depression. In addition, a more recent study found that having a depressed mother increased the probability of adolescent anxiety and depression, as well as the amount of peer stress these adolescents experienced [30].

National restrictions on harmful pesticides were thought to be one of the most effective ways to avoid self-poisoning suicide [5, 6]. However, this intervention had relatively less impact on preventing suicide by self-poisoning, and toxic substances such as pesticides were still readily accessible among many countries, including China. In addition, Weerasinghe et al. [31] showed that pesticide vendors were willing to be a “gatekeeper” to prevent patient’s access to pesticides from their stores. Nonetheless, it was challenging for the sellers to differentiate between consumers who might be self-poisoning and those who bought pesticides for agricultural use [32]. Thus, the matter at hand is complicated. Even worse, illicit drugs and medically prescribed drugs are also readily accessible and the most frequently misused medicine [7, 8]. But one was sure that suicides was able to, at least partially, be decreased by limiting the accessibility of means to suicide [24], by training hospital doctors and community medical workers to screen people at high risk of suicide, manage self-poisoning patients more appropriately, as well as provide adequate follow-up health care [24]. Suicidality is a significant social and medical issue, and hence it should be prioritized in many areas.

### Limitations

This study still needs to be improved in a few areas. Initially, a longitudinal cohort study is required to investigate because it is challenging to establish a causal relationship between variables and suicide due to the cross-sectional study’s nature. Secondly, the meaning of the term “suicide” in the literature that is currently available is occasionally unclear, which may result in patient’s selection bias. On the other hand, for the current study, we gathered patients who had been admitted to the hospital’s department for self-poisoning suicide. Thus, those patients are highly specific. Thirdly, as the data were gathered from a medical center, certain possible biases may still remain as a result of unmeasured confounding variables. A planned multicenter validation study is therefore necessary.

## Conclusions

Self-poisoning suicide patients have severe mental health issues. Patients who self-poison have a close connection to their family member’s mental health, particularly their levels of anxiety and depression. According to the findings, being married and adopting healthy lifestyle habits, such as quitting smoking and drinking, increasing their physical activity levels, and managing their idle time, are able to help patients with mental health concerns and even suicidal thoughts.

## Supplementary Information

**Additional file 1: Supplementary table 1.** Measures of mental health among patients and their family members.

**Additional file 2: Supplementary table 2.** Multivariable analysis of significant characteristics for predicting self-poisoning suicide among patients after adjusting for age and gender (*n*=151).

**Additional file 3: Supplementary table 3**. Multivariable analysis of significant characteristics in terms of matched family members for predicting self-poisoning suicide among patients after adjusting for age and gender (*n*=151).

**Additional file 4: Supplementary table 4**. Multivariable analysis of significant characteristics for predicting anxiety among self-poisoning suicide patients after adjusting for age and gender (*n*=102).

**Additional file 5: Supplementary table 5**. Multivariable analysis of significant characteristics for predicting depression among self-poisoning suicide patients after adjusting for age and gender (*n*=102).

**Additional file 6: Supplementary table 6**. Multivariable analysis of significant characteristics in terms of matched family members for predicting anxiety among self-poisoning suicide patients after adjusting for age and gender (*n*=102).

**Additional file 7: Supplementary table 7**. Multivariable analysis of significant characteristics from matched family members for predicting depression among self-poisoning suicide patients after adjusting for age and gender (*n*=102).

## Data Availability

The data are available under reasonable request to the corresponding author.

## Abbreviations

BHS-20: Beck hopelessness scale-20
CI: Confident interval
COVID-19: Corona Virus Disease 2019
GAD-7: Generalized anxiety disorder-7
LL: Lower limit; OR, Odds ratio
PHQ-9: Patient health questionnaire-9
SD: Standard deviation
SES-10: Self-esteem scale-10
Std. Error: Standard error
SSQ-10: Social support questionnaire-10
UL: Upper limit

## References

[1] Albano GD, Malta G, La Spina C, Rifiorito A, Provenzano V, Triolo V (2022). Toxicological Findings of Self-Poisoning Suicidal Deaths: A Systematic Review by Countries. Toxics.

[2] Phillips MR, Yang G, Zhang Y, Wang L, Ji H, Zhou M (2002). Risk factors for suicide in China: a national case-control psychological autopsy study. Lancet.

[3] Spiller HA, Ackerman JP, Spiller NE, Casavant MJ (2019). Sex- and Age-specific Increases in Suicide Attempts by Self-Poisoning in the United States among Youth and Young Adults from 2000 to 2018. J Pediatr.

[4] Kasemy ZA, Sharif AF, Amin SA, Fayed MM, Desouky DE, Salama AA (2022). Trend and epidemiology of suicide attempts by self-poisoning among Egyptians. PLoS ONE.

[5] Gunnell D, Knipe D, Chang SS, Pearson M, Konradsen F, Lee WJ (2017). Prevention of suicide with regulations aimed at restricting access to highly hazardous pesticides: a systematic review of the international evidence. Lancet Glob Health.

[6] Gunnell D, Eddleston M, Phillips MR, Konradsen F (2007). The global distribution of fatal pesticide self-poisoning: systematic review. BMC Public Health.

[7] VujaklijaBrajkovic A, Grgat M, Bielen L, Brajkovic J, Zlopasa O, Vrdoljak NG (2022). Self-poisoning as a cause of admission in a medical intensive care unit and a question of misuse of prescription medications. Heart Lung.

[8] Karaca O, Ertaskin A (2020). Epidemiology of Self-poisoning with Drug in the Central Anatolian Region in Turkey. Cureus.

[9] Pushpakumara PHGJ, Dawson AH, Adikari AMP, Thennakoon SUB, Abeysinghe R, Rajapakse TN (2021). Exploration of associations between deliberate self-poisoning and psychiatric disorders in rural Sri Lanka: A case-control study. PLoS ONE.

[10] Thumtecho S, Sriworasuwat P, Wainipitapong S (2022). Suicidal attempts and self-poisoning: 1-year retrospective cohort study from the quaternary hospital in Thai metropolitan area. Health Sci Rep.

[11] Chai Y, Luo H, Yip PSF (2020). Prevalence and risk factors for repetition of non-fatal self-harm in Hong Kong, 2002–2016: A population-based cohort study. Lancet Reg Health West Pac.

[12] Gobbi G, Atkin T, Zytynski T (2019). Association of Cannabis Use in Adolescence and Risk of Depression, Anxiety, and Suicidality in Young Adulthood: A Systematic Review and Meta-analysis. Jama Psychiat.

[13] Pawer S, Rajabali F, Zheng A, Smith J, Purssell R, Pike I (2021). Analyses of Child and Youth Self-Poisoning Hospitalizations by Substance and Socioeconomic Status. Int J Environ Res Public Health.

[14] Thaysa Bier de Sousa N, Vedana KGG, Zanetti ACG, de Souza J, da Silva AHS, Miasso AI. Intentional self-poisoning with medications: Occurrence, recurrence and suicide deaths. Death Stud. 2023:1–9. https://www.tandfonline.com/doi/abs/10.1080/07481187.2023.2175390?journalCode=udst20. 10.1080/07481187.2023.2175390 36794403

[15] Knipe D, Silva T, Aroos A, Senarathna L, Hettiarachchi NM, Galappaththi SR (2021). Hospital presentations for self-poisoning during COVID-19 in Sri Lanka: an interrupted time-series analysis. Lancet Psychiatry.

[16] Zhou Y, Xu J, Rief W (2020). Are comparisons of mental disorders between Chinese and German students possible? An examination of measurement invariance for the PHQ-15, PHQ-9 and GAD-7. BMC Psychiatry.

[17] Zachurzok A, Pasztak-Opilka A, Gawlik AM. Depression, anxiety and self-esteem in adolescent girls with polycystic ovary syndrome. Ginekol Pol. 2021. https://journals.viamedica.pl/ginekologia_polska/article/view/71910. 10.5603/GP.a2021.0042 33751507

[18] Bouvard M, Charles S, Guerin J, Aimard G, Cottraux J. Study of Beck’s hopelessness scale Validation and factor analysis. Encephale. 1992;18(3):237–40. 1299593

[19] Fu C, Wang GW, Shi XX, Cao FL (2021). Social support and depressive symptoms among physicians in tertiary hospitals in China: a cross-sectional study. BMC Psychiatry.

[20] Quintana JM, Gonzalez N, Bilbao A, Aizpuru F, Escobar A, Esteban C (2006). Predictors of patient satisfaction with hospital health care. BMC Health Serv Res.

[21] Zeng X, Lu M, Chen M (2021). The relationship between family intimacy and relapse tendency among people who use drugs: a moderated mediation model. Subst Abuse Treat Prev Policy.

[22] Turecki G, Brent DA (2016). Suicide and suicidal behaviour. Lancet.

[23] Wang B, Han L, Wen J, Zhang J, Zhu B (2020). Self-poisoning with pesticides in Jiangsu Province, China: a cross-sectional study on 24,602 subjects. BMC Psychiatry.

[24] Bachmann S (2018). Epidemiology of Suicide and the Psychiatric Perspective. Int J Environ Res Public Health.

[25] Hu N, Glauert RA, Li JH, Taylor CL (2016). Risk factors for repetition of a deliberate self-harm episode within seven days in adolescents and young adults: A population-level record linkage study in Western Australia. Aust Nz J Psychiat.

[26] Pellegrini L, Maietti E, Rucci P, Casadei G, Maina G, Fineberg NA (2020). Suicide attempts and suicidal ideation in patients with obsessive-compulsive disorder: A systematic review and meta-analysis. J Affect Disord.

[27] Stanley IH, Boffa JW, Rogers ML, Hom MA, Albanese BJ, Chu C (2018). Anxiety sensitivity and suicidal ideation/suicide risk: A meta-analysis. J Consult Clin Psychol.

[28] Wiebenga JX, Eikelenboom M, Heering HD, van Oppen P, Penninx BW (2021). Suicide ideation versus suicide attempt: Examining overlapping and differential determinants in a large cohort of patients with depression and/or anxiety. Aust N Z J Psychiatry.

[29] Yap MB, Pilkington PD, Ryan SM, Jorm AF (2014). Parental factors associated with depression and anxiety in young people: a systematic review and meta-analysis. J Affect Disord.

[30] Henry LM, Steele EH, Watson KH, Bettis AH, Gruhn M, Dunbar J (2020). Stress Exposure and Maternal Depression as Risk Factors for Symptoms of Anxiety and Depression in Adolescents. Child Psychiat Hum D.

[31] Weerasinghe M, Pearson M, Peiris R, Dawson AH, Eddleston M, Jayamanne S (2014). The role of private pesticide vendors in preventing access to pesticides for self-poisoning in rural Sri Lanka. Inj Prev.

[32] Konradsen F, Hoek W, Peiris P. Reaching for the bottle of pesticide–a cry for help. Self-inflicted poisonings in Sri Lanka. Soc Sci Med. 2006;62(7):1710–9. 10.1016/j.socscimed.2005.08.020 16165259

