# Supplementary material for: Association of mental health status between self-poisoning suicide patients and their family members: a matched-pair analysis
Source: BMC Psychiatry. 2023 Apr 28;23:294. doi: 10.1186/s12888-023-04779-9 (PMC10144897; doi:10.1186/s12888-023-04779-9)
Supplement: Supplementary file 1 — Additional file 1: Supplementary table 1. Measures of mental health among patients and their family members. [file 12888_2023_4779_MOESM1_ESM.docx]

| **Supplementary table 1.** Measures of mental health among patients and their family members. | | | | | |
| --- | --- | --- | --- | --- | --- |
| **Features** | **Measures** | **Score range** | **Indications** | **Cronbach’s α** | **References** |
| Anxiety | Generalized anxiety disorder-7 (GAD-7) | 0 to 21 | Positive correlation  Score indicates a more serious anxious status | 0.953 | [1] |
| Depression | Patient health questionnaire-9 (PHQ-9) | 0 to 27 | Positive correlation  higher scores indicating severer depressive conditions | 0.960 | [1] |
| Self-esteem | Self-esteem scale-10 (SES-10) | 10 to 40 | A higher SES-10 score indicates better self-esteem. | 0.782 | [2] |
| Hopelessness status | Beck hopelessness scale-20 (BHS-20) | 0 to 20 | higher scores representing severer hopelessness | 0.784 | [3] |
| Social support status | Social support questionnaire-10 (SSQ-10) | 8 to 70 | A higher score of SSQ-10 indicates better social support. | 0.823 | [4] |

**References**

1. Zhou Y, Xu J, Rief W. Are comparisons of mental disorders between Chinese and German students possible? An examination of measurement invariance for the PHQ-15, PHQ-9 and GAD-7. Bmc Psychiatry. 2020 Oct 1;20(1). PMID: WOS:000576960600001. doi: ARTN 480

10.1186/s12888-020-02859-8.

2. Zachurzok A, Pasztak-Opilka A, Gawlik AM. Depression, anxiety and self-esteem in adolescent girls with polycystic ovary syndrome. Ginekol Pol. 2021 Mar 10. PMID: 33751507. doi: 10.5603/GP.a2021.0042.

3. Bouvard M, Charles S, Guerin J, Aimard G, Cottraux J. [Study of Beck's hopelessness scale. Validation and factor analysis]. Encephale. 1992 May-Jun;18(3):237-40. PMID: 1299593.

4. Fu C, Wang GW, Shi XX, Cao FL. Social support and depressive symptoms among physicians in tertiary hospitals in China: a cross-sectional study. Bmc Psychiatry. 2021 Apr 29;21(1). PMID: WOS:000654888800004. doi: ARTN 217

10.1186/s12888-021-03219-w.
