# Supplementary material for: Association of mental health status between self-poisoning suicide patients and their family members: a matched-pair analysis
Source: BMC Psychiatry. 2023 Apr 28;23:294. doi: 10.1186/s12888-023-04779-9 (PMC10144897; doi:10.1186/s12888-023-04779-9)
Supplement: Supplementary file 2 — Additional file 2: Supplementary table 2. Multivariable analysis of significant characteristics for predicting self-poisoning suicide among patients after adjusting for age and gender (n=151). [file 12888_2023_4779_MOESM2_ESM.docx]

| **Supplementary table 2**. Multivariable analysis of significant characteristics for predicting self-poisoning suicide among patients after adjusting for age and gender (n=151). | | | | |
| --- | --- | --- | --- | --- |
| **Characteristics** | **OR** | **95% CI** | | **P** |
|  |  | **LL** | **UL** |  |
| (Intercept) | 1.95 | 1.55 | 2.46 | 0.000 |
| Gender |  |  |  |  |
| Male | Ref. |  |  |  |
| Female | 1.13 | 1.01 | 1.27 | 0.037 |
| Age | 1.00 | 0.99 | 1.00 | 0.579 |
| Marital status |  |  |  |  |
| Single | Ref. |  |  |  |
| Dating | 0.81 | 0.64 | 1.02 | 0.078 |
| Married | 0.84 | 0.69 | 1.01 | 0.062 |
| Divorced or widowed | 0.79 | 0.59 | 1.05 | 0.101 |
| Smoking |  |  |  |  |
| Yes | Ref. |  |  |  |
| No | 0.77 | 0.67 | 0.89 | 0.000 |
| Sedentary time (hours) |  |  |  |  |
| Less than 1 | Ref. |  |  |  |
| 1~3 | 0.85 | 0.74 | 0.98 | 0.023 |
| 3~6 | 0.83 | 0.71 | 0.98 | 0.028 |
| Above 6 | 0.84 | 0.71 | 0.99 | 0.040 |
| Sport frequency per week |  |  |  |  |
| 0 | Ref. |  |  |  |
| 1~2 | 1.20 | 1.04 | 1.39 | 0.014 |
| 3~5 | 1.04 | 0.89 | 1.22 | 0.640 |
| Above 5 | 0.80 | 0.66 | 0.96 | 0.017 |
| Severity of anxiety (GAD-7) ^a^ |  |  |  |  |
| None | Ref. |  |  |  |
| Mild | 1.46 | 1.24 | 1.72 | 0.000 |
| Moderate | 1.89 | 1.61 | 2.22 | 0.000 |
| Severe | 1.63 | 1.37 | 1.94 | 0.000 |
| OR, Odds ratio; CI, Confident interval; LL, Lower limit; UL, Upper limit; GAD-7, Generalized anxiety disorder-7.  ^a^ none anxiety indicates a GAD-7 or PHQ-9 score of 0 to 4, mild anxiety indicates a score of 5 to 9, moderate anxiety indicates a score of 10 to 14, and severe anxiety indicates a score of 15 or above. | | | | |
