# Supplementary material for: Association of mental health status between self-poisoning suicide patients and their family members: a matched-pair analysis
Source: BMC Psychiatry. 2023 Apr 28;23:294. doi: 10.1186/s12888-023-04779-9 (PMC10144897; doi:10.1186/s12888-023-04779-9)
Supplement: Supplementary file 3 — Additional file 3: Supplementary table 3. Multivariable analysis of significant characteristics in terms of matched family members for predicting self-poisoning suicide among patients after adjusting for age and gender (n=151). [file 12888_2023_4779_MOESM3_ESM.docx]

| **Supplementary table 3**. Multivariable analysis of significant characteristics in terms of matched family members for predicting self-poisoning suicide among patients after adjusting for age and gender (n=151). | | | | |
| --- | --- | --- | --- | --- |
| **Characteristics** | **OR** | **95% CI** | | **P** |
|  |  | **LL** | **UL** |  |
| (Intercept) | 3.97 | 2.58 | 6.12 | 0.000 |
| Family relationship |  |  |  |  |
| Parents | Ref. |  |  |  |
| Spouse | 0.66 | 0.56 | 0.79 | 0.000 |
| Siblings | 0.80 | 0.53 | 1.21 | 0.293 |
| Kids | 0.82 | 0.63 | 1.05 | 0.121 |
| Others | 0.74 | 0.51 | 1.06 | 0.104 |
| Gender |  |  |  |  |
| Male | Ref. |  |  |  |
| Female | 0.77 | 0.65 | 0.91 | 0.003 |
| Age | 0.99 | 0.98 | 1.00 | 0.053 |
| Education level |  |  |  |  |
| Primary | Ref. |  |  |  |
| High school | 1.41 | 1.16 | 1.71 | 0.001 |
| University | 0.96 | 0.80 | 1.15 | 0.668 |
| Graduate | 0.80 | 0.57 | 1.13 | 0.210 |
| Personality |  |  |  |  |
| Outgoing | Ref. |  |  |  |
| Middle | 0.91 | 0.77 | 1.07 | 0.236 |
| Introvert | 1.30 | 1.02 | 1.65 | 0.038 |
| Unclear | 0.86 | 0.63 | 1.16 | 0.319 |
| OR, Odds ratio; CI, Confident interval; LL, Lower limit; UL, Upper limit. | | | | |
