# Supplementary material for: Association of mental health status between self-poisoning suicide patients and their family members: a matched-pair analysis
Source: BMC Psychiatry. 2023 Apr 28;23:294. doi: 10.1186/s12888-023-04779-9 (PMC10144897; doi:10.1186/s12888-023-04779-9)
Supplement: Supplementary file 4 — Additional file 4: Supplementary table 4. Multivariable analysis of significant characteristics for predicting anxiety among self-poisoning suicide patients after adjusting for age and gender (n=102). [file 12888_2023_4779_MOESM4_ESM.docx]

| **Supplementary table 4**. Multivariable analysis of significant characteristics for predicting anxiety among self-poisoning suicide patients after adjusting for age and gender (n=102). | | | | |
| --- | --- | --- | --- | --- |
| **Characteristics** | **OR** | **95% CI** | | **P** |
|  |  | **LL** | **UL** |  |
| (Intercept) | 29.56 | 16.52 | 52.88 | 0.000 |
| Gender |  |  |  |  |
| Male | Ref. |  |  |  |
| Female | 1.26 | 0.89 | 1.77 | 0.196 |
| Age | 1.00 | 0.98 | 1.02 | 0.967 |
| Marital status |  |  |  |  |
| Single | Ref. |  |  |  |
| Dating | 0.73 | 0.37 | 1.41 | 0.347 |
| Married | 0.43 | 0.25 | 0.75 | 0.003 |
| Divorced or widowed | 3.43 | 1.55 | 7.56 | 0.003 |
| Sedentary time (hours) |  |  |  |  |
| Less than 1 | Ref. |  |  |  |
| 1~3 | 1.36 | 0.92 | 2.01 | 0.125 |
| 3~6 | 0.73 | 0.45 | 1.19 | 0.206 |
| Above 6 | 1.20 | 0.70 | 2.07 | 0.506 |
| Monthly income (￥) |  |  |  |  |
| Less than 3000 | Ref. |  |  |  |
| 3000~6000 | 0.87 | 0.58 | 1.30 | 0.489 |
| 6000~9000 | 4.44 | 2.08 | 9.50 | 0.000 |
| Above 9000 | 4.28 | 1.25 | 14.61 | 0.023 |
| History of psychiatry disease |  |  |  |  |
| Yes | Ref. |  |  |  |
| No | 0.45 | 0.31 | 0.65 | 0.000 |
| OR, Odds ratio; CI, Confident interval; LL, Lower limit; UL, Upper limit. | | | | |
