# Supplementary material for: Association of mental health status between self-poisoning suicide patients and their family members: a matched-pair analysis
Source: BMC Psychiatry. 2023 Apr 28;23:294. doi: 10.1186/s12888-023-04779-9 (PMC10144897; doi:10.1186/s12888-023-04779-9)
Supplement: Supplementary file 5 — Additional file 5: Supplementary table 5. Multivariable analysis of significant characteristics for predicting depression among self-poisoning suicide patients after adjusting for age and gender (n=102). [file 12888_2023_4779_MOESM5_ESM.docx]

| **Supplementary table 5**. Multivariable analysis of significant characteristics for predicting depression among self-poisoning suicide patients after adjusting for age and gender (n=102). | | | | |
| --- | --- | --- | --- | --- |
| **Characteristics** | **OR** | **95% CI** | | **P** |
|  |  | **LL** | **UL** |  |
| (Intercept) | 39.70 | 19.48 | 80.92 | 0.000 |
| Gender |  |  |  |  |
| Male | Ref. |  |  |  |
| Female | 1.42 | 0.98 | 2.06 | 0.063 |
| Age | 1.03 | 1.01 | 1.05 | 0.013 |
| Marital status |  |  |  |  |
| Single | Ref. |  |  |  |
| Dating | 1.09 | 0.52 | 2.30 | 0.821 |
| Married | 0.21 | 0.12 | 0.39 | 0.000 |
| Divorced or widowed | 1.44 | 0.62 | 3.37 | 0.402 |
| Drinking |  |  |  |  |
| Yes | Ref. |  |  |  |
| No | 0.67 | 0.42 | 1.09 | 0.109 |
| Monthly income (￥) |  |  |  |  |
| Less than 3000 | Ref. |  |  |  |
| 3000~6000 | 0.87 | 0.59 | 1.29 | 0.492 |
| 6000~9000 | 4.66 | 2.11 | 10.32 | 0.000 |
| Above 9000 | 2.90 | 0.82 | 10.20 | 0.100 |
| History of psychiatry disease |  |  |  |  |
| Yes | Ref. |  |  |  |
| No | 0.45 | 0.30 | 0.68 | 0.000 |
| OR, Odds ratio; CI, Confident interval; LL, Lower limit; UL, Upper limit. | | | | |
