# Supplementary material for: Association of mental health status between self-poisoning suicide patients and their family members: a matched-pair analysis
Source: BMC Psychiatry. 2023 Apr 28;23:294. doi: 10.1186/s12888-023-04779-9 (PMC10144897; doi:10.1186/s12888-023-04779-9)
Supplement: Supplementary file 6 — Additional file 6: Supplementary table 6. Multivariable analysis of significant characteristics in terms of matched family members for predicting anxiety among self-poisoning suicide patients after adjusting for age and gender (n=102). [file 12888_2023_4779_MOESM6_ESM.docx]

| **Supplementary table 6**. Multivariable analysis of significant characteristics in terms of matched family members for predicting anxiety among self-poisoning suicide patients after adjusting for age and gender (n=102). | | | | |
| --- | --- | --- | --- | --- |
| **Characteristics** | **OR** | **95% CI** | | **P** |
|  |  | **LL** | **UL** |  |
| (Intercept) | 24.66 | 9.15 | 66.47 | 0.000 |
| Gender |  |  |  |  |
| Male | Ref. |  |  |  |
| Female | 1.36 | 0.91 | 2.02 | 0.141 |
| Age | 0.98 | 0.96 | 1.01 | 0.136 |
| Education level |  |  |  |  |
| Primary | Ref. |  |  |  |
| High school | 1.74 | 1.10 | 2.77 | 0.021 |
| University | 0.84 | 0.49 | 1.45 | 0.544 |
| Graduate | 1.07 | 0.34 | 3.41 | 0.910 |
| Monthly income (￥) |  |  |  |  |
| Less than 3000 | Ref. |  |  |  |
| 3000~6000 | 0.97 | 0.65 | 1.43 | 0.867 |
| 6000~9000 | 2.13 | 1.09 | 4.16 | 0.029 |
| Above 9000 | 2.00 | 1.05 | 3.81 | 0.037 |
| Personality |  |  |  |  |
| Outgoing | Ref. |  |  |  |
| Middle | 0.68 | 0.45 | 1.04 | 0.081 |
| Introvert | 1.48 | 0.85 | 2.59 | 0.172 |
| Unclear | 0.38 | 0.16 | 0.88 | 0.027 |
| Severity of anxiety (GAD-7) ^a^ |  |  |  |  |
| None | Ref. |  |  |  |
| Mild | 0.38 | 0.22 | 0.66 | 0.001 |
| Moderate | 2.22 | 1.25 | 3.93 | 0.008 |
| Severe | 2.67 | 1.00 | 7.16 | 0.054 |
| OR, Odds ratio; CI, Confident interval; LL, Lower limit; UL, Upper limit; GAD-7, Generalized anxiety disorder-7.  ^a^ none anxiety indicates a GAD-7 or PHQ-9 score of 0 to 4, mild anxiety indicates a score of 5 to 9, moderate anxiety indicates a score of 10 to 14, and severe anxiety indicates a score of 15 or above. | | | | |
