# Supplementary material for: Association of mental health status between self-poisoning suicide patients and their family members: a matched-pair analysis
Source: BMC Psychiatry. 2023 Apr 28;23:294. doi: 10.1186/s12888-023-04779-9 (PMC10144897; doi:10.1186/s12888-023-04779-9)
Supplement: Supplementary file 7 — Additional file 7: Supplementary table 7. Multivariable analysis of significant characteristics from matched family members for predicting depression among self-poisoning suicide patients after adjusting for age and gender (n=102). [file 12888_2023_4779_MOESM7_ESM.docx]

| **Supplementary table 7**. Multivariable analysis of significant characteristics from matched family members for predicting depression among self-poisoning suicide patients after adjusting for age and gender (n=102). | | | | |
| --- | --- | --- | --- | --- |
| **Characteristics** | **OR** | **95% CI** | | **P** |
|  |  | **LL** | **UL** |  |
| (Intercept) | 4.93 | 1.61 | 15.08 | 0.006 |
| Gender |  |  |  |  |
| Male | Ref. |  |  |  |
| Female | 1.57 | 1.02 | 2.42 | 0.044 |
| Age | 1.01 | 0.99 | 1.03 | 0.338 |
| Sport frequency per week |  |  |  |  |
| 0 | Ref. |  |  |  |
| 1~2 | 2.67 | 1.26 | 5.66 | 0.012 |
| 3~5 | 3.34 | 1.45 | 7.68 | 0.006 |
| Above 5 | 4.33 | 1.93 | 9.71 | 0.001 |
| Personality |  |  |  |  |
| Outgoing | Ref. |  |  |  |
| Middle | 0.61 | 0.39 | 0.95 | 0.031 |
| Introvert | 1.59 | 0.89 | 2.86 | 0.122 |
| Unclear | 0.64 | 0.26 | 1.58 | 0.336 |
| Severity of anxiety (GAD-7) ^a^ |  |  |  |  |
| None | Ref. |  |  |  |
| Mild | 0.33 | 0.16 | 0.68 | 0.003 |
| Moderate | 0.61 | 0.23 | 1.61 | 0.322 |
| Severe | 0.51 | 0.16 | 1.64 | 0.264 |
| Severity of depression (PHQ-9) ^a^ |  |  |  |  |
| None | Ref. |  |  |  |
| Mild | 2.00 | 0.99 | 4.04 | 0.057 |
| Moderate | 1.30 | 0.59 | 2.87 | 0.512 |
| Severe | 3.51 | 1.17 | 10.50 | 0.027 |
| OR, Odds ratio; CI, Confident interval; LL, Lower limit; UL, Upper limit; GAD-7, Generalized anxiety disorder-7; PHQ-9, Patient health questionnaire-9.  ^a^ none anxiety or depression indicates a GAD-7 or PHQ-9 score of 0 to 4, mild anxiety or depression indicates a score of 5 to 9, moderate anxiety or depression indicates a score of 10 to 14, and severe anxiety or depression indicates a score of 15 or above. | | | | |
